# Alzheimer’s disease as a causal risk factor for diabetic retinopathy: a Mendelian randomization study

**DOI:** 10.3389/fendo.2024.1340608

**Published:** 2024-04-18

**Authors:** Fu Ouyang, Ping Yuan, Yaxin Ju, Wei Chen, Zijun Peng, Hongbei Xu

**Affiliations:** ^1^ Department of Neurology, the Affiliated Hospital of Guizhou Medical University, Guiyang, Guizhou, China; ^2^ Department of Neurology, The First Affiliated Hospital of Chongqing Medical University, Chongqing, China

**Keywords:** Alzheimer’s disease, diabetic retinopathy, Mendelian randomization, FUMA, causal association

## Abstract

**Objectives:**

This study aims to investigate the causal relationship between Alzheimer’s Disease (AD) and Diabetic Retinopathy (DR).

**Methods:**

Employing Mendelian Randomization (MR), Generalized Summary-data-based Mendelian Randomization (GSMR), and the MR-Steiger test, this study scrutinizes the genetic underpinnings of the hypothesized causal association between AD and DR, as well as its Proliferative DR (PDR) and Non-Proliferative DR (NPDR) subtypes. Comprehensive data from Genome-Wide Association Studies (GWAS) were analyzed, specifically AD data from the Psychiatric Genomics Consortium (71,880 cases/383,378 controls), and DR, PDR, and NPDR data from both the FinnGen consortium (FinnGen release R8, DR: 5,988 cases/314,042 controls; PDR: 8,383 cases/329,756 controls; NPDR: 3,446 cases/314,042 controls) and the IEU OpenGWAS (DR: 14,584 cases/176,010 controls; PDR: 8,681 cases/204,208 controls; NPDR: 2,026 cases/204,208 controls). The study also incorporated Functional Mapping and Annotation (FUMA) for an in-depth analysis of the GWAS results.

**Results:**

The MR analyses revealed that genetic susceptibility to AD significantly increases the risk of DR, as evidenced by GWAS data from the FinnGen consortium (OR: 2.5090; 95% confidence interval (CI):1.2102-5.2018, false discovery rate P-value (*P_FDR_*)=0.0201; GSMR: b_xy_=0.8936, b_xy_se_=0.3759, *P*=0.0174), NPDR (OR: 2.7455; 95% CI: 1.3178-5.7197, *P_FDR_* =0.0166; GSMR: b_xy_=0.9682, b_xy_se_=0.3802, *P*=0.0126), and PDR (OR: 2.3098; 95% CI: 1.2411-4.2986, *P_FDR_* =0.0164; GSMR: b_xy_=0.7962, b_xy_se_=0.3205, *P*=0.0129) using DR GWAS from FinnGen consortium. These results were corroborated by DR GWAS datasets from IEU OpenGWAS. The MR-Steiger test confirmed a significant association of all identified instrumental variables (IVs) with AD. While a potential causal effect of DR and its subtypes on AD was identified, the robustness of these results was constrained by a low power value. FUMA analysis identified OARD1, NFYA, TREM1 as shared risk genes between DR and AD, suggesting a potential genetic overlap between these complex diseases.

**Discussion:**

This study underscores the contribution of AD to an increased risk of DR, as well as NPDR and PDR subtypes, underscoring the necessity of a holistic approach in the management of patients affected by these conditions.

## 1 Introduction

Alzheimer’s Disease (AD), the most common form of dementia, is a progressively worsening neurodegenerative condition. Characterized by neurotic plaques and neurofibrillary tangles, AD results from the accumulation of amyloid-beta peptide (Aβ) in the brain (1). Diabetic Retinopathy (DR), a common microvascular complication of Diabetes Mellitus (DM), is a leading cause of preventable vision loss in the elderly (2). According to the Global Burden of Disease Study, DR is the fifth primary cause of blindness and moderate to severe visual impairment in adults over 50 (3). Early signs of Non-Proliferative Diabetic Retinopathy (NPDR) include vascular endothelial damage, microaneurysm formation, and dot intraretinal hemorrhages. Increasing ischemia can lead to Proliferative DR (PDR), which poses a high risk of vision loss due to complications like vitreous hemorrhage or retinal detachment, as blood vessels grow into the vitreous (4). Previous studies have suggested shared genetic risk factors between AD and DR, with extensive epidemiological research exploring this potential link (5–7). However, the direct causal relationship between AD, DR, and its subtypes NPDR and PDR, remains unclear, partly due to confounding factors.

Mendelian Randomization (MR) is a statistical methodology that aids in exploring cause-effect relationships between variables by leveraging genetic variations that influence the exposure of interest (8). Recently, MR has become a powerful tool for assessing causal relationships in epidemiology and genetics. By using genetic variants as instrumental variables, MR provides evidence of causality that is less prone to bias from confounding factors and reverse causation (9). We further used Generalized Summary-data-based Mendelian Randomization (GSMR), which excludes SNPs that demonstrate pleiotropic effects, enhances the confidence of the MR results (10). Moreover, MR-Steiger filtering was used to ensure that the IVs were strongly correlated with the exposure rather than the outcome (11). Additionally, the Functional Mapping and Annotation (FUMA) method, a widely-used computational tool, integrates genetic association data with functional annotations, gene expression data, and pathway analyses (12). This integration helps identify relevant genetic variants and their possible biological mechanisms. In our study, we apply both MR and FUMA methods to uncover new insights into the potential cause-effect relationship between AD and DR. Our approach also enhances the existing knowledge base about the shared genetic underpinnings of these conditions and sheds light on the biological mechanisms that may underlie this connection.

## 2 Methods

### 2.1 Study design and data source

**Figure 1:**
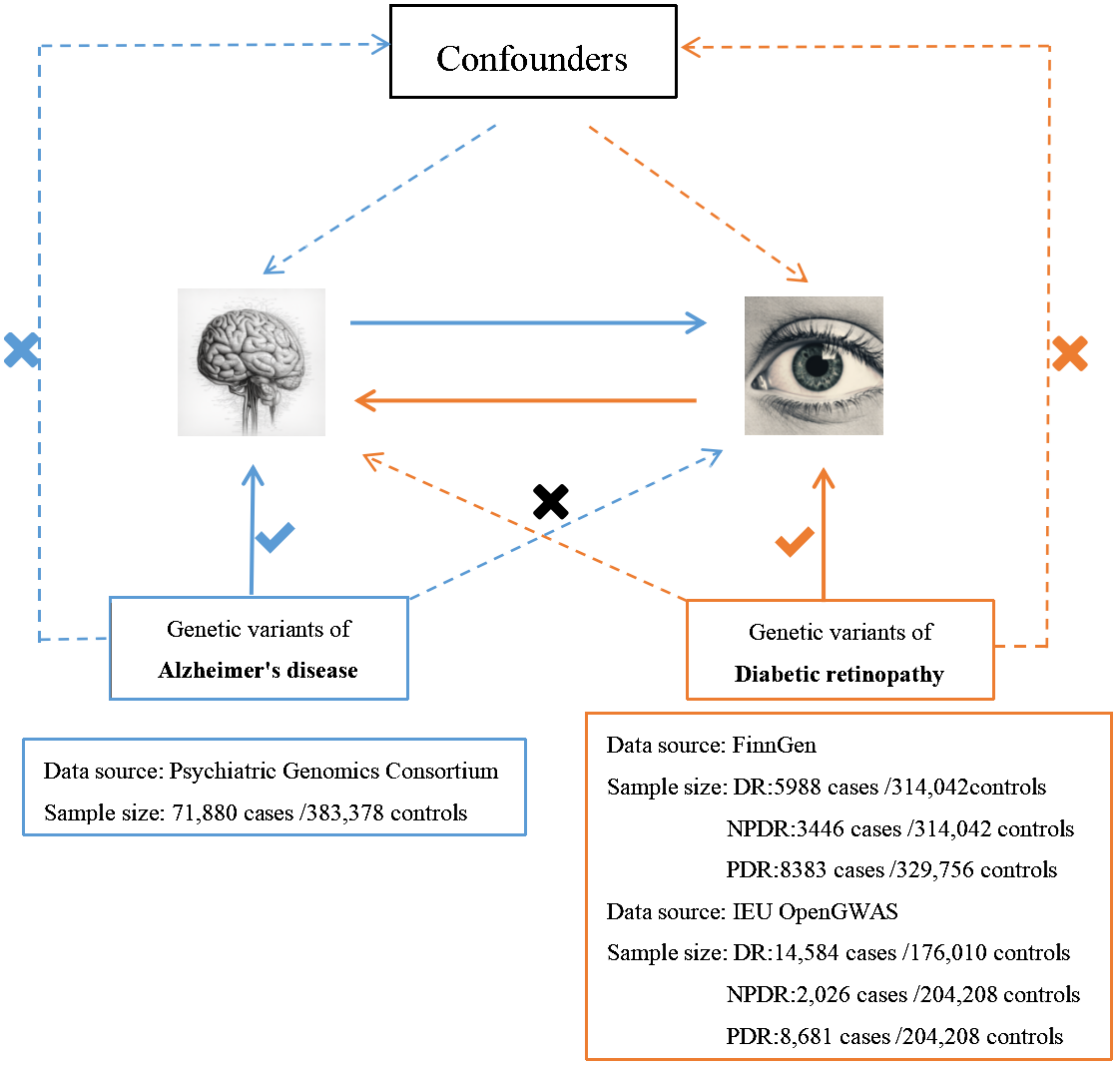
An overview of the study design.

We employed the MR technique in this study, which is an instrumental variable (IVs) analysis leveraging genetic variants like single-nucleotide polymorphisms (SNPs) as exposure proxies. To validate the chosen SNPs as IVs, three core assumptions must be met: (1) the Association assumption, asserting the relevance of SNPs with the exposure; (2) the Independence assumption, which holds that genetic variants affect outcomes solely via their impact on exposure, excluding other causal pathways; (3) the Exclusion assumption, requiring the genetic variants to be conditionally independent of the outcome given the exposure and confounders (13). **Figure 1** illustrates the schematic diagram of this MR study. The AD dataset used was derived from a large-scale Genome-Wide Association Study (GWAS) provided by the Psychiatric Genomics Consortium. The datasets for DR, PDR, and NPDR were gathered from two sources: the FinnGen consortium (FinnGen release R8) and the IEU OpenGWAS database, respectively. **Table 1** summarizes these datasets.

**Table 1:** Detailed information of the studies and datasets used for Mendelian randomization analysis.

Phenotype	Sample size (cases/controls)	Population	Consortium	Year	Journal
AD	71,880/383,378	Mixed	Psychiatric Genomics Consortium	2019	Nat Genet
DR	5988/314,042	Mixed	FinnGen	2022	–
PDR	8383/329,756	Mixed	FinnGen	2022	–
NPDR	3446/314,042	Mixed	FinnGen	2022	–
DR	14,584/176,010	European	IEU OpenGWAS	2021	–
PDR	8,681/204,208	European	IEU OpenGWAS	2021	–
NPDR	2,026/204,208	European	IEU OpenGWAS	2021	–

DR, diabetic retinopathy; PDR, proliferative diabetic retinopathy; NPDR, background diabetic retinopathy; AD, alzheimer's disease.

### 2.2 Selection of IVs

The IVs were selected for AD and each DR subtype. This selection was based on the hypothesis that the IVs must exhibit a robust correlation with the exposure. SNP IVs were initially identified from the Genome-Wide Association Studies (GWAS) summary statistics related to exposure, with the correlation threshold relaxed to *P*<5×10^−6^ *(* 14). Subsequently, a clumping algorithm was employed to choose independent SNP IVs, applying an r^2^ threshold of 0.001 within a 5000 kb linkage disequilibrium (LD) window (15). This approach effectively reduced the risk of overestimating the number of independent tests (16). SNPs that demonstrated an F value exceeding 10 in the GWAS of exposure were chosen as potential IVs, thereby diminishing the risk of weak instrument bias (17). To eliminate potential horizontal pleiotropy, SNPs directly associated with outcomes or confounding factors were excluded (18). SNPs significantly linked to confounders were sourced from the Phennoscanner database (http://www.phenoscanner.medschl.cam.ac.uk/). R^2^ and F-statistic for each SNP were calculated using the formulas R^2^ = 2×EAF×(1-EAF)×β^2^ and F=(β)^2^/(SE)^2^ respectively (19). The specific characteristics and F value of selected SNPs were presented in **Supplementary Tables S1, S2**.

### 2.3 MR analysis

A bidirectional two-sample MR analysis was conducted using the TwoSampleMR R package (20) and the MR-PRESSO package (21), both powerful tools for inferring the potential causal relationship between exposure and outcome. The forward MR analysis considered AD as the exposure and each DR subtype as the outcome, while the reverse analysis switched these roles. The primary MR analysis employed the inverse-variance weighted (IVW) method within a random effects model to evaluate the causal impact of exposure on the outcome (22). The IVW method combines SNP-specific causal estimates to obtain a weighted average, considering the inverse of the variance of these estimates. To test the robustness of our findings, four supplementary MR methods were used: weighted median, MR-Egger method, weighted mode, and simple mode (23). Each of these methods is designed to address various forms of pleiotropy and potential biases, offering a comprehensive view of the potential causal relationship. In cases where only a single IV SNP is available, the Wald ratio method was used (24). Multiple testing adjustments and false discovery rate (FDR) corrected *P*-value calculations were performed using the Benjamini-Hochberg method. This approach effectively minimizes the risk of false-positive outcomes arising from multiple comparisons.

### 2.4 Generalized summary-data-based Mendelian randomization analysis

To estimate credible causal associations using the IVW regression method in MR analysis, we incorporated the GSMR estimates. Implemented via the GSMR R package, this method evaluates causal associations (b_xy_=b_zx_/b_zy_) between a risk factor (b_zx_) and an outcome (b_zy_), utilizing summary-level data from GWASs. In this context, z represents the genotype of an SNP (coded as 0, 1, or 2), x denotes the exposure in standard deviation (SD) units, and y signifies the outcome on the logit scale(logarithm of the odds ratio, logOR). The method calculates b_zy_ as the effect of z on y on the logit scale, b_zx_ as the effect of z on x, and b_xy_ as the effect of x on y, free from confounding by non-genetic factors. Additionally, GSMR accounts for LD between multiple correlated SNPs used as IVs and excludes SNPs that demonstrate pleiotropic effects, as evidenced by the heterogeneity in dependent instruments outlier analysis (HEIDI-outlier test <0.01) (10).

### 2.5 The analysis of MR Steiger and MR Steiger filtering

Furthermore, we employed MR Steiger and MR Steiger filtering, as implemented in the TwoSampleMR R package, to investigate the causal direction between exposure and outcome. This methodology calculates the variance explained in both exposure and outcome by the instrumental SNPs and tests whether the variance in the outcome is less than that in the exposure. The MR Steiger directionality test determines the validity of the chosen SNPs as IVs. A “TRUE” MR Steiger result implies causality in the anticipated direction, whereas a “FALSE” result indicates causality in the opposite direction. SNPs yielding “FALSE” results were excluded, signifying their primary effect on outcomes rather than exposures (11). Finally, the mRnd online power calculator (https://shiny.cnsgenomics.com/mRnd/) was utilized for power calculation (25), a critical step to ensure the study’s design is sufficiently robust to discern the causal estimate.

### 2.6 Sensitivity analysis

In the sensitivity analysis, the leave-one-out approach was used to determine the impact of individual SNPs on the study outcomes. To confirm the robustness of the second and third core MR assumptions, Cochran’s Q statistic and the MR-Egger regression method were applied. These methods played a key role in identifying heterogeneity and pleiotropy (20). Particularly, the MR-Egger regression was crucial for assessing the potential effects of directional pleiotropy on risk estimates via intercept tests. Recognizing the possible limitations of the MR-Egger method, the MR Pleiotropy Residual Sum and Outlier (MR-PRESSO) test was also implemented. This approach proved essential in pinpointing outlier SNPs and evaluating potential horizontal pleiotropy (26).

### 2.7 Statistical analyses

Statistical analyses were performed using R software, version 4.2.1. In the MR analysis, associations with *P* values below 0.05 (including both raw *P* and *P_FDR_* =0.05) were deemed too strongly indicate causal relationships. In contrast, associations exhibiting raw *P* values under 0.05 but with *P_FDR_* exceeding 0.05 were considered to provide only suggestive evidence of associations.

### 2.8 Analysis of FUMA

The current investigation employed FUMA platform, an integrative, web-based tool that leverages a multitude of biological data sources. This platform was instrumental in the functional annotation of GWAS results, the prioritization of genes, and facilitating interactive visualization (12). Gene and gene-set analyses were performed using the Multi-marker Analysis of GenoMic Annotation (MAGMA) version 1.6, seamlessly integrated within FUMA (27). MAGMA facilitates multi-marker analysis by correlating gene-level statistics with GWAS summary statistics, offering a gene-centric view of genetic associations. We imported a comprehensive analysis of preprocessed GWAS data for AD and each DR subtype into FUMA 1.5.3. SNPs were considered independently significant if they had a P-value below 5×10^-8^ and an r^2^ value under 0.6 in the GWAS results. Among these significant independent SNPs, lead SNPs were determined based on a pairwise r^2^ below 0.1. Our analysis further identified genomic risk loci containing SNPs in strong LD (r2>0.6) with these independently significant SNPs. These lead SNPs are crucial in capturing the genetic association signal specific to each locus. We continued this analysis by pinpointing genomic risk loci that included SNPs in robust LD with the significant independent SNPs. LD blocks were merged into a single genomic locus if they were within a 250 kb range. For these LD analyses, we used reference genetic data from European populations, as outlined in the 1000 Genomes Project phase 3 database. Finally, SNPs with functional annotations were linked to specific genes. Notably, protein-coding genes were pinpointed through positional mapping, expression quantitative trait loci (eQTL) mapping, and chromatin interaction mapping (28). These methods are crucial for linking genetic variants to specific, functionally relevant genes.

## 3 Results

### 3.1 Genetically predicted AD with DR, NPDR and PDR

**Figure 2:**
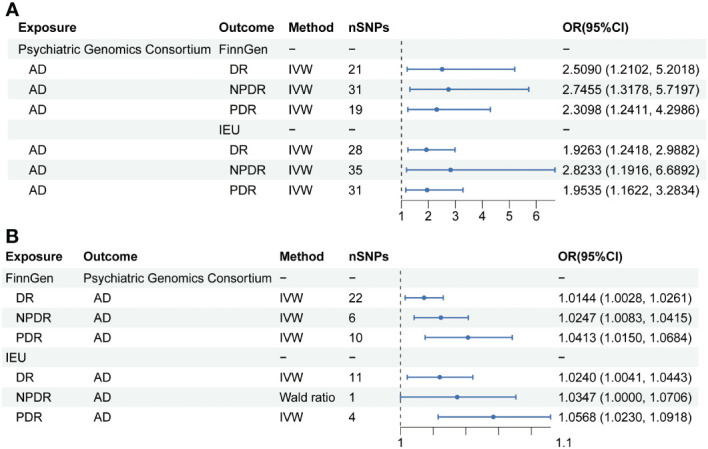
Mendelian randomization results of causal effects between AD and DR. **(A)** MR analysis with AD as exposure. **(B)** MR analysis with DR, NPDR and PDR as exposure.

In exploring the causal relationship of AD on DR, AD was utilized as the exposure, while DR and its subtypes, as identified in the FinnGen GWAS database, were considered the outcomes. For AD, a total of 21, 19, and 31 SNPs were selected, respectively. The IVW method revealed that genetically predisposed AD causally led to a 1.509-fold increase in the risk of DR(OR 2.5090; 95% CI: 1.2102-5.2018, *P*=0.0134, *P_FDR_* =0.0201, power=99%), a 1.3098-fold increase in the risk of PDR (OR 2.3098; 95% CI: 1.2411-4.2986, *P*=0.0082, *P_FDR_* =0.0164, power=99%), and a 1.7455-fold increase in the risk of NPDR (OR 2.7455; 95% CI: 1.3178-5.7197, *P*=0.0069, *P_FDR_* =0.0166, power=100%). Moreover, sensitivity analysis employing the GSMR method corroborated these associations (DR: b_xy_=0.8936, b_xy_se_=0.3759, *P*=0.0174; PDR: b_xy_=0.7962, b_xy_se_=0.3205, *P*=0.0129; NPDR: b_xy_=0.9682, b_xy_se_=0.3802, *P*=0.0126) (**Table 2**, **Figure 2**).

**Table 2:** Main results of the Mendelian randomization analysis.

Exposure	Outcome	IVW						GSMR			
		nSNPs	Method	OR (95% CI)	P value	P_FDR_	Power	bxy	bxy_se	bxy_pval	OR (95% CI)
DR and its subtypes database from FinnGen consortium											
AD	DR	21	IVW	2.5090 (1.2102, 5.2018)	0.0134	0.0201	99%	0.8936	0.3759	0.0174	2.44 (1.17, 5.11)
	PDR	19	IVW	2.3098 (1.2411, 4.2986)	0.0082	0.0164	99%	0.7962	0.3205	0.0129	2.22 (1.18, 0.16)
	NPDR	31	IVW	2.7455 (1.3178, 5.7197)	0.0069	0.0166	100%	0.9682	0.3802	0.0126	2.58 (1.23, 5.44)
DR and its subtypes database from IEU OpenGWAS											
AD	DR	28	IVW	1.9263 (1.2418, 2.9882)	0.0034	0.0102	99%	0.6039	0.2279	0.0080	1.83 (1.17, 2.86)
	PDR	31	IVW	1.9535 (1.1622, 3.2834)	0.0115	0.0197	98%	0.6051	0.2696	0.0248	1.83 (1.08, 3.11)
	NPDR	35	IVW	2.8233 (1.1916, 6.6892)	0.0184	0.0201	99%	0.4404	0.3650	0.2276	1.55 (0.76, 3.18)
DR and its subtypes database from FinnGen consortium											
DR	AD	22	IVW	1.0144 (1.0028, 1.0261)	0.0150	0.02	20%	0.0134	0.0055	0.0147	1.01 (1.00, 1.02)
PDR		10	IVW	1.0413 (1.0150, 1.0684)	0.0020	0.012	41%	0.0193	0.0133	0.1472	1.02 (0.99, 1.05)
NPDR		6	IVW	1.0247 (1.0083, 1.0415)	0.0031	0.0124	25%	0.0202	0.0094	0.0313	1.02 (1.00, 1.04)
DR and its subtypes database from IEU OpenGWAS											
DR	AD	11	IVW	1.0240 (1.0041, 1.0443)	0.0176	0.0211	15%	0.0107	0.0103	0.2956	1.01 (0.99, 1.03)
PDR		4	IVW	1.0568 (1.0230, 1.0918)	0.0008	0.0096	36%	–	–	–	–
NPDR		1	Wald ratio	1.0347 (1.0000, 1.0706)	0.0497	0.0497	17%	–	–	–	–

nSNPs, number of single-nucleotide polymorphisms; IVW, inverse-variance weighted; OR, odds ratio; CI, confidence interval; GSMR, Generalized Summary-data-based Mendelian Randomization; FDR, false discovery rate; DR, diabetic retinopathy; PDR, proliferative diabetic retinopathy; NPDR, background diabetic retinopathy; AD, alzheimer's disease.

Further analysis using the IEU openGWAS database, which included 28 SNPs for AD, 35 for NPDR, and 31 for PDR, supported these observations. Both the IVW and GSMR analyses demonstrated a significant link between AD and an increased risk of DR (OR 1.9263; 95% CI: 1.2418-2.9882, *P*=0.0034, *P_FDR_* =0.0102, power=99%; GSMR: b_xy_=0.6039, b_xy_se_=0.2279, *P*=0.0080), NPDR (OR 2.8233; 95% CI: 1.1916-6.6892, *P*=0.0184, *P_FDR_* =0.0201, power=99%; GSMR: b_xy_=0.4404, b_xy_se_=0.3650, *P*=0.2276), and PDR (OR 1.9535, 95% CI: 1.1622-3.2834, *P*=0.0115, *P_FDR_* =0.0197, power=98%; GSMR: b_xy_=0.6051, b_xy_se_=0.2696, *P*=0.0248) (**Table 2**, **Figure 2**). Additional results from four other MR methods - the weighted median, MR-Egger, weighted mode, and simple mode - are provided in the **Supplementary Table S3**. Lastly, the MR Steiger test validated the SNP selection, affirming the hypothesized causal direction of AD’s impact on DR (**Table 3**).

**Table 3:** Main results of the bi-directional MR-Steiger test.

Exposure	Outcome	nSNPs		MR analysis (all SNPs)		MR analysis (valid SNPs)	
		Total	Invaild	OR IVW (95% CI)	P value	OR IVW (95% CI)	P value
DR and its subtypes database from FinnGen consortium							
AD	DR	21	0	2.5090 (1.2102, 5.2018)	0.0134	2.5090 (1.2102, 5.2018)	0.0134
	PDR	19	0	2.3098 (1.2411, 4.2986)	0.0082	2.3098 (1.2411, 4.2986)	0.0082
	NPDR	31	0	2.7455 (1.3178, 5.7197)	0.0069	2.7455 (1.3178, 5.7197)	0.0069
DR and its subtypes database from IEU OpenGWAS							
AD	DR	28	0	1.9263 (1.2418, 2.9882)	0.0034	1.9263 (1.2418, 2.9882)	0.0034
	PDR	31	0	1.9535 (1.1622, 3.2834)	0.0115	1.9535 (1.1622, 3.2834)	0.0115
	NPDR	35	0	2.8233 (1.1916, 6.6892)	0.0184	2.8233 (1.1916, 6.6892)	0.0184
DR and its subtypes database from FinnGen consortium							
DR	AD	22	0	1.0144 (1.0028, 1.0261)	0.0150	1.0144 (1.0028, 1.0261)	0.0150
PDR		10	0	1.0413 (1.0150, 1.0684)	0.0020	1.0413 (1.0150, 1.0684)	0.0020
NPDR		6	0	1.0247 (1.0083, 1.0415)	0.0031	1.0247 (1.0083, 1.0415)	0.0031
DR and its subtypes database from IEU OpenGWAS							
DR	AD	11	0	1.0240 (1.0041, 1.0443)	0.0176	1.0240 (1.0041, 1.0443)	0.0176
PDR		4	0	1.0568 (1.0230, 1.0918)	0.0008	1.0568 (1.0230, 1.0918)	0.0008
NPDR		1	0	1.0347 (1.0000, 1.0706)	0.0497	1.0347 (1.0000, 1.0706)	0.0497

### 3.2 No causal association of DR, NPDR and PDR on AD

In the reverse MR analysis, AD was considered the outcome, with 22 and 11 SNPs included for DR, 6 and 1 SNPs for NPDR, as well as 10 and 4 SNPs for PDR, sourced from the FinnGen and IEU openGWAS databases, respectively. Utilizing the FinnGen GWAS database, no conclusive causal effects of DR (OR 1.0144, 95% CI: 1.0028-1.0261, *P*=0.0150, *P_FDR_* =0.02, power=20%; GSMR: b_xy_=0.0134, b_xy_se_=0.0055, *P*=0.0147), NPDR (OR 1.0247, 95% CI: 1.0083-1.0415, *P*=0.0031, *P_FDR_* =0.0124, power=25%; GSMR: b_xy_=0.0202, b_xy_se_=0.0094, *P*=0.0313), or PDR (OR 1.0413, 95% CI: 1.0150-1.0684, *P*=0.0020, *P_FDR_* =0.012, power=41%; GSMR: b_xy_=0.0193, b_xy_se_=0.0133, *P*=0.1472) on AD were established, primarily due to low statistical power. This uncertainty persisted when employing the IEU OpenGWAS dataset. Here, no definitive causal effects were found for DR (OR 1.0240, 95% CI: 1.0041-1.0443, *P*=0.0176, *P_FDR_* =0.0211, power=15%; GSMR: bxy=0.0107, bxy_se=0.0103, *P*=0.2956), PDR (OR 1.0568, 95% CI: 1.0230-1.0918, *P*=0.0008, *P_FDR_* =0.0096, power=36%), and NPDR (OR 1.0347, 95% CI: 1.0000-1.0706, *P*=0.0497, *P_FDR_* =0.0497, power=17%), attributed to the notably low power values. Furthermore, due to a limited number of SNPs, GSMR analysis was not conducted for causal estimation of PDR or NPDR on AD. The MR-Steiger test further substantiated these results by confirming the absence of horizontal pleiotropy among the SNPs (**Table 3**). Details of the power calculations are provided in the **Supplementary Table S4**.

### 3.3 Sensitivity analysis

The bidirectional MR analysis revealed an absence of pleiotropy, as evidenced by the non-significant MR-Egger intercept. Further, the analysis demonstrated a lack of heterogeneity, corroborated by Cochran’s Q statistic (**Table 4**). The leave-one-out method indicated that the causal association remained robust with the exclusion of each individual SNP (**Supplementary Figure S1**), suggesting no influential SNPs. Additionally, the funnel plot, scatter plot, and forest plot showed no significant outliers. The effect estimate of each SNP on AD and DR is visualized in the forest plot, displayed in **Supplementary Figures S2-S12**.

**Table 4:** Heterogeneity and pleiotropy tests for the associations of AD and DR.

Exposure	outcome	Cochrane’s *Q* test		MR-Egger intercept test		MRPRESSO global test
		Q-value	PQ	Intercept	P intercept	P value
DR and its subtypes database from FinnGen consortium						
AD	DR	6.9966	0.9967	0.0014	0.9272	0.998
	PDR	7.4207	0.9861	0.0110	0.5313	0.989
	NPDR	23.2106	0.8065	0.0124	0.4839	0.828
DR and its subtypes database from IEU OpenGWAS						
AD	DR	20.2689	0.8193	0.0078	0.4266	0.824
	PDR	28.3745	0.5506	0.0104	0.3673	0.536
	NPDR	26.4170	0.8202	0.0162	0.4531	0.812
DR and its subtypes database from FinnGen consortium						
DR	AD	25.0194	0.2463	-0.0026	0.1807	0.279
PDR		5.4445	0.7940	0.0020	0.7765	0.781
NPDR		2.9638	0.7056	0.0016	0.7113	0.759
DR and its subtypes database from IEU OpenGWAS						
DR	AD	3.2225	0.9757	0.0019	0.5241	0.975
PDR		4.2235	0.2383	0.0041	0.6538	0.378
NPDR		–	–	–	–	–

DR, diabetic retinopathy; PDR, proliferative diabetic retinopathy; NPDR, background diabetic retinopathy; AD, alzheimer's disease.

### 3.4 Post-genome-wide association study annotation by FUMA

**Figure 3:**
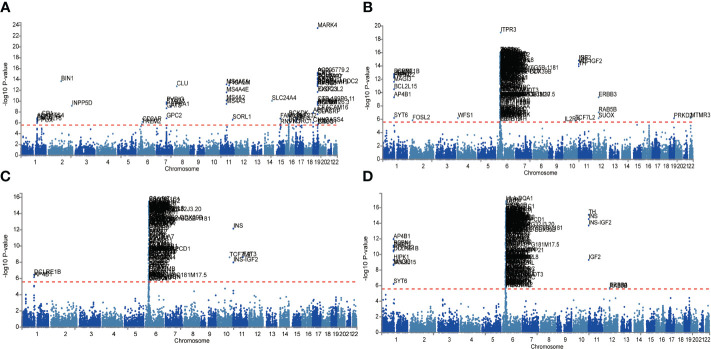
Manhattan plot of the gene-based test as computed by MAGMA based on input GWAS summary statistics of AD **(A)**, DR **(B)**, NPDR **(C)** and PDR **(D)**. Genome wide significance (red dashed line in the plot) was defined at P = 0.05/N.

Given the larger total sample size of the FinnGen consortium compared to the IEU openGWAS, along with a similar control sample size as the AD GWAS data, the FUMA analysis was conducted using the FinnGen consortium database to ensure more accurate results. The Manhattan plot of the input GWAS summary statistics and the gene-based test, as computed by MAGMA based on input GWAS summary statistics, is provided in **Figure 3**.

**Figure 4:**
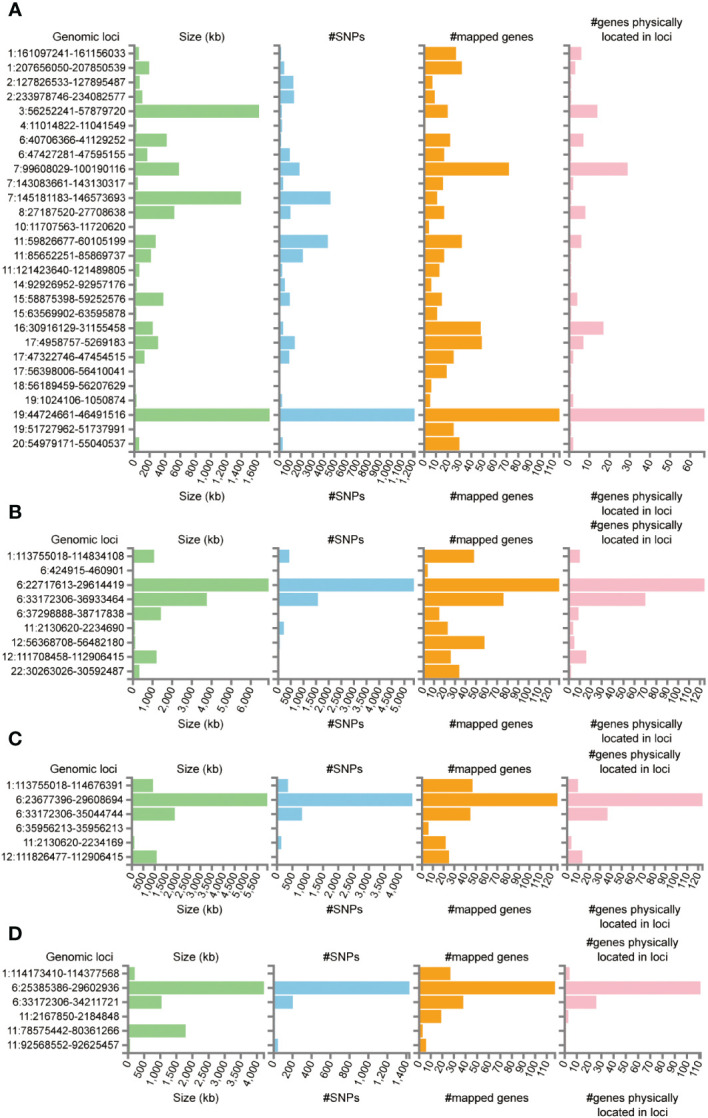
Genetic risk loci identified by FUMA analysis. **(A)** Genetic risk loci for AD. **(B)** Genetic risk loci for DR. **(C)** Genetic risk loci for NPDR. **(D)** Genetic risk loci for PDR. Genomic risk loci was displayed in the format of “chromosome: start position–end position”. Each genomic locus was represented by a series of histograms, arranged from left to right to display the size of the locus, the number of candidate SNPs, the number of mapped genes, and the number of known genes located within it.

Functional annotation of the summary statistics of AD and DR GWAS databases using the FUMA platform identified 252 independent significant SNPs and 94 lead SNPs across 28 genomic risk loci for AD. In addition, for DR, 9 genomic risk loci, 461 independent significant SNPs, and 145 lead SNPs were identified. For NPDR, there were 307 independent significant SNPs and 94 lead SNPs identified, along with 6 genomic risk loci, and for PDR, 79 independent significant SNPs, 29 lead SNPs, and 6 genomic risk loci were detected (**Figure 4**; **Supplementary Tables S5-S8**). Furthermore, 666 protein-coding risk genes were identified for AD, 408 for DR, 270 for NPDR, and 210 for PDR (**Supplementary Table S9**).

**Figure 5:**
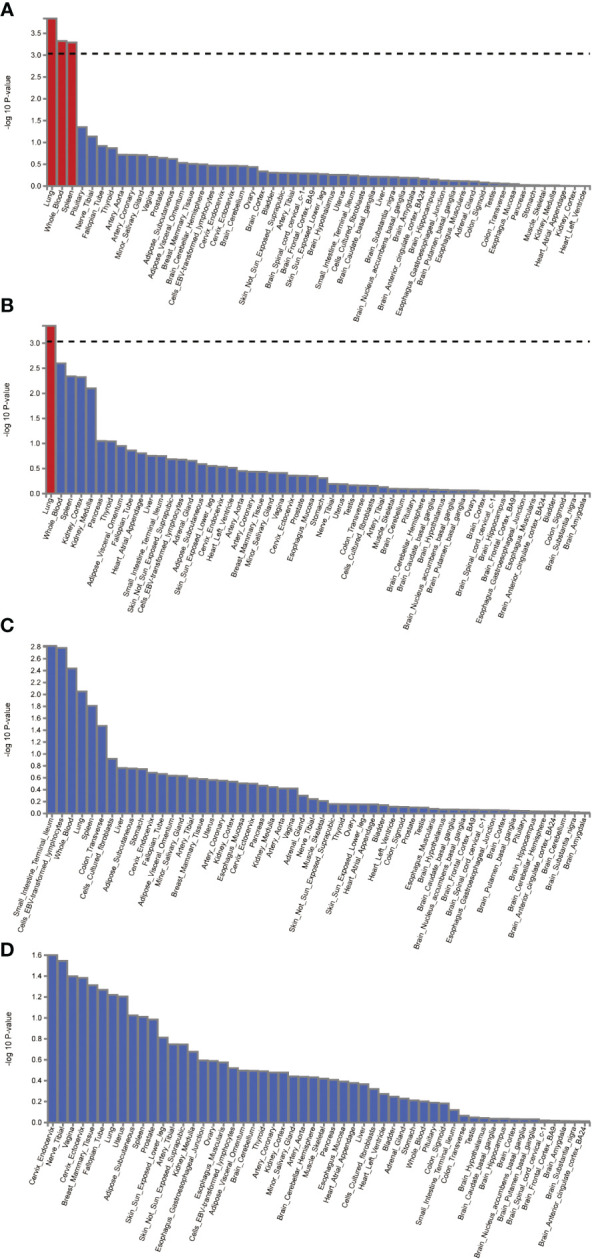
The analysis of tissue enrichment by FUMA using GTEx v8 (n=54 tissues). **(A)** Tissue enrichment for AD. **(B)** Tissue enrichment for DR. **(C)** Tissue enrichment for NPDR. **(D)** Tissue enrichment for PDR.

The FUMA platform enabled the conduct of MAGMA tissue-specificity analysis on 54 tissues obtained from GTEx V8 (**Figure 5**). Our findings suggest a nominally significant association between AD, DR, PDR, and NPDR with gene associations in whole blood, brain regions, and the pituitary (*P*<0.05). Further analysis on tissue-specific expression highlighted significant enrichment of AD GWAS hits in lung tissue, whole blood, and the spleen, while DR GWAS hits were primarily enriched in the lung. Interestingly, no substantial tissue enrichment was detected for NPDR and PDR. In subsequent investigations, we focused on 13 brain regions and the pituitary, discovering that disease-gene associations related to AD and DR were enriched in brain areas such as the pituitary, brain cortex, hippocampus, and cerebellum. Through this in-depth analysis, we identified four differentially expressed genes common between AD, DR, NPDR, and PDR: OARD1, NFYA, CHI3L2, and CD48. A noteworthy observation was the down-regulation of OARD1, NFYA, and CD48, while CHI3L2 was up-regulated. Importantly, we identified OARD1, NFYA, and TREM1 as shared risk genes between AD and DR. These risk genes were all found to be located on Chromosome 6, providing intriguing insights into the potential genetic intersection of these complex diseases (**Supplementary Table S10**, **Supplementary Figure S13**).

## 4 Discussion

The potential causative link between DR and AD has been a focal point in epidemiological and clinical research fields. In our current study, we utilized a bidirectional MR approach, offering a comprehensive perspective on this causal relationship. Our results strongly indicate that AD may contribute to DR, including both NPDR and PDR. However, the influence of DR on AD, while evident, showed a lesser degree of association, calling for careful interpretation of these findings. Shared risk factors, identified via FUMA analysis, such as OARD1, NFYA, and TREM1, point to potential biological pathways connecting these two disorders.

AD and DR are complex conditions presenting significant global public health concerns. The complex factors underlying the association between DR and AD risks remain to be fully clarified. AD patients with cognitive impairments have exhibited specific retinal changes, including amyloid plaque formation (29, 30), neuronal loss (31), and optic neuropathy (32). Human retinal autopsies have revealed concurrent hyperphosphorylated tau and Aβ accumulation, key indicators of AD in the brain (33, 34). Early retinal neurovascular abnormalities could potentially act as markers for future cognitive decline (35). Given the remarkable similarity between the microvasculature of the retina and brain, changes in retinal blood vessels may indirectly reflect similar alterations in the cerebral microvasculature (36). The accelerated cognitive aging associated with diabetes might partly stem from the combined effects of blood-brain barrier disruption and/or ischemic damage, leading to various brain tissue changes (36). Crucially, our study endorses the view of AD as a causal risk factor for DR, as well as both NPDR and PDR, as deduced from our highly reliable MR methodology.

Previous research has acknowledged DR as a significant risk factor for dementia (36–38). Patients with DM and DR have a 34% increased risk of developing AD compared to those without DR (38), positioning DR as a potential key biomarker for dementia risk, alongside glycemia and renal complications (39). However, this association must be considered with caution due to potential bias from non-random missing data. Another cohort study, involving 29,961 individuals with Type 2 DM, found that sight-threatening DR was associated with an increased risk of incident dementia, even after adjusting for vascular risk factors and diabetes severity (37). This analysis, though, did not account for DR duration or APOE genotype and relied on ICD codes for dementia diagnoses, rather than expert consensus or research criteria (39). In our study, we identified a potential causal link between DR and AD. However, due to the relatively low power value of our findings, they should be interpreted cautiously and warrant further investigation.

The study revealed a stronger association between AD and NPDR compared to AD and PDR in both the FinnGen and IEU datasets. DR, a complication of diabetes, progresses through two stages: NPDR and PDR (40). NPDR, the initial stage, is characterized by mild microvascular changes and often presents minimal symptoms (41). These changes mainly result from inflammatory responses under hyperglycemic conditions and direct glucose-induced damage to retinal microcirculation. Progression to PDR is typically influenced by factors such as poor blood sugar control and increased VEGF expression, leading to pre-retinal neovascularization (42). Interestingly, AD is characterized by enhanced immune responses and microglial activation, which contribute to neurodegeneration (36). There is growing evidence that the neuroinflammatory mechanisms in AD are similar to those in DR (5). Considering these similarities, the study suggests a closer link between AD and NPDR than with PDR. However, further research is necessary to more clearly understand these relationships and mechanisms.

Our study identified three shared genetic risk factors between DR and AD - OARD1, NFYA, and TREM1 - using FUMA analysis. These factors offer intriguing avenues for understanding the biological interactions between these diseases and may shed light on potential shared disease mechanisms. OARD1 (O-acetyl-ADP-ribose deacetylase 1) is a gene involved in the metabolism of O-acetyl-ADP-ribose, a molecule implicated in cellular processes like DNA repair (43) and cell cycle progression (44, 45). Considering that both AD and DR involve disturbances in cellular homeostasis and integrity, OARD1’s association with these diseases could reflect disruptions in DNA repair or cell cycle regulation. NFYA (Nuclear Transcription Factor Y Subunit Alpha) regulates various genes associated with cellular growth and differentiation (46–48). Dysregulation in these processes could lead to the pathological changes seen in both AD and DR, including neuronal degeneration and abnormal angiogenesis, respectively. TREM1 (Triggering receptor expressed on myeloid cells 1) is an immunoglobulin superfamily transmembrane protein (49) related to immune response and inflammation (50). Studies have shown increased TREM1 expression in microglia around amyloid-beta plaques in AD mice, and inhibiting TREM1 could alleviate neuroinflammation and amyloid-beta pathology (51). Moreover, genetic variations in TREM1 are linked to an increased risk of AD (52). Altered immune responses and cell interactions, implicated in both DR and AD, may contribute to chronic inflammation, neuronal loss, and capillary degeneration. While our study identified these shared risk factors, the exact role of each gene in AD and DR pathogenesis remains to be fully understood. Investigating how these genes contribute to the diseases’ development and progression at a molecular level is crucial. Future experimental studies on these genes could provide valuable insights into the shared pathological mechanisms between DR and AD, highlighting the intricate relationship between metabolic, immune, and neurodegenerative processes and the multidimensional nature of these diseases.

Despite these findings, our study has certain limitations. First, the MR analysis demonstrated a low power value in establishing the causal relationship between DR and AD. This issue might stem from an insufficient cohort sample size and the limited availability of suitable datasets enriched with comprehensive genetic and phenotypic information. These factors could affect the statistical robustness and reliability of our results. Second, the selection of instrumental variables (IVs) for MR analysis might not have been optimal, potentially due to confounding factors. Third, the role of genetic predisposition in disease outcomes could be limited, as genetic factors only contribute a small portion to the total variance in complex diseases like AD and DR. Fourth, environmental and behavioral factors play a significant role in the development and progression of DR, along with genetic factors (53). Major risk factors include the duration of diabetes, levels of glycated hemoglobin (HbA1c), and blood pressure. High HbA1c levels, indicative of poor blood glucose control, can damage retinal microvessels and lead to DR (44). Effective blood glucose management is essential to reduce DR risks and its progression. Hypertension also contributes to DR by exacerbating microvascular damage (54). Additionally, harmful lifestyle choices such as smoking and alcohol consumption increase DR risks (55, 56). While these factors are crucial, the genetic aspect of DR development shouldn’t be overlooked (57). Lastly, although our study identified OARD1, NFYA, and TREM1 as shared risk factors, the exact role of these genes in the development and progression of AD and DR at the molecular level is still unclear. Future research focusing on these genes could offer valuable insights into the shared pathological mechanisms between DR and AD.

## 5 Conclusions

In conclusion, the extensive MR study’s results robustly support the theory that AD significantly contributes to the development of DR. Furthermore, the study discovered common risk genes, suggesting a potential link between these two intricate diseases. These findings emphasize the possibility that targeting AD could be an effective therapeutic strategy to slow down DR’s pathological progression. Consequently, this warrants further exploration within a clinical setting.

## Data availability statement

The datasets presented in this study can be found in online repositories. The names of the repository/repositories and accession number(s) can be found in the article/**Supplementary Material**.

## Ethics statement

The MR analysis used in this study relies on summary data obtained from prior studies, all of which had secured appropriate ethical approval and informed consent. Given that our study did not engage directly with any participants, it did not necessitate additional ethical clearance for this secondary analysis. This method ensures that we abide by the tenets of ethical research while efficiently leveraging available data resources.

## Author contributions

FO: Writing – original draft. PY: Writing – original draft. YJ: Writing – original draft, Data curation, Formal analysis, Methodology. WC: Writing – original draft, Formal analysis, Methodology. ZP: Writing – original draft, Formal analysis, Validation. HX: Conceptualization, Supervision, Validation, Writing – review & editing.
