# Supplementary material for: Alzheimer’s disease as a causal risk factor for diabetic retinopathy: a Mendelian randomization study
Source: Front Endocrinol (Lausanne). 2024 Apr 18;15:1340608. doi: 10.3389/fendo.2024.1340608 (PMC11064697; doi:10.3389/fendo.2024.1340608)
Supplement: Supplementary file 1 [file DataSheet_1.zip › Supplementary Table S1-4, Fig S1-13.docx]

**Supplementary**

**Table S1. Characteristics of selected SNPs for DR and its subtypes database**

| **SNP** | **Trait** | **Chr** | **Pos.** | **Effect allele** | **Other allele** | **EAF** | **Beta** | **SE** | **P value** | **R2** | **F statistic** |
| --- | --- | --- | --- | --- | --- | --- | --- | --- | --- | --- | --- |
| DR and its subtypes database from FinnGen consortium | | | | | | | | | | | |
| rs11074369 | DR | 16 | 19159095 | A | G | 0.369876 | 0.0900382 | 0.0193463 | 3.26E-06 | 0.003778903 | 21.65996609 |
| rs11102605 | DR | 1 | 113248884 | C | T | 0.366905 | 0.0894322 | 0.0191906 | 3.16E-06 | 0.003715697 | 21.7175446 |
| rs111273527 | DR | 9 | 14855363 | T | C | 0.0157961 | 0.322712 | 0.0681513 | 2.19E-06 | 0.003238137 | 22.42239199 |
| rs114785141 | DR | 2 | 179814616 | T | C | 0.00604119 | 0.510208 | 0.103732 | 8.72E-07 | 0.00312619 | 24.1918469 |
| rs141413577 | DR | 6 | 38061327 | A | G | 0.00804113 | 0.457621 | 0.0916903 | 6.01E-07 | 0.003340817 | 24.90950539 |
| rs144536316 | DR | 6 | 27466315 | A | G | 0.0290362 | 0.441871 | 0.0481509 | 4.44E-20 | 0.011009404 | 84.21358997 |
| rs147392974 | DR | 6 | 27331812 | A | G | 0.0297151 | -0.404638 | 0.0619965 | 6.72E-11 | 0.009441474 | 42.59895902 |
| rs1819138 | DR | 6 | 160705042 | T | C | 0.165096 | 0.117806 | 0.0248481 | 2.13E-06 | 0.003825938 | 22.47752286 |
| rs1987903 | DR | 8 | 15464913 | A | G | 0.188976 | -0.118004 | 0.0243151 | 1.22E-06 | 0.004268387 | 23.55273457 |
| rs28636873 | DR | 15 | 57397943 | C | A | 0.4952 | 0.0976671 | 0.0185891 | 1.49E-07 | 0.004768992 | 27.60449825 |
| rs402072 | DR | 19 | 46715865 | C | T | 0.139345 | -0.140757 | 0.0277486 | 3.92E-07 | 0.004752154 | 25.73107027 |
| rs4884126 | DR | 13 | 79235278 | G | A | 0.26288 | 0.0982327 | 0.021021 | 2.97E-06 | 0.00373971 | 21.8376257 |
| rs57454310 | DR | 20 | 419185 | A | G | 0.106741 | 0.143858 | 0.0293304 | 9.36E-07 | 0.003946451 | 24.05647912 |
| rs61688654 | DR | 12 | 14364805 | C | A | 0.0751219 | 0.159319 | 0.0341681 | 3.12E-06 | 0.003527087 | 21.74169929 |
| rs72697239 | DR | 1 | 119936184 | T | C | 0.0583039 | 0.192607 | 0.0375888 | 2.99E-07 | 0.004073639 | 26.25591822 |
| rs72698768 | DR | 14 | 100838538 | G | A | 0.146479 | -0.135658 | 0.0272527 | 6.43E-07 | 0.004601616 | 24.77831382 |
| rs7317962 | DR | 13 | 30280325 | T | G | 0.529176 | 0.091085 | 0.0188113 | 1.29E-06 | 0.004134114 | 23.44531684 |
| rs74203920 | DR | 21 | 44294411 | T | C | 0.0369013 | 0.28167 | 0.0457647 | 7.52E-10 | 0.00563928 | 37.88087028 |
| rs77342925 | DR | 20 | 61050137 | C | A | 0.13674 | -0.130923 | 0.0278734 | 2.64E-06 | 0.004046682 | 22.06236179 |
| rs78342400 | DR | 1 | 244871801 | A | G | 0.190982 | 0.120625 | 0.0237538 | 3.81E-07 | 0.0044963 | 25.78745373 |
| rs7899394 | DR | 10 | 65118057 | C | T | 0.782058 | 0.104967 | 0.022964 | 4.86E-06 | 0.003755912 | 20.89346615 |
| rs79014846 | DR | 2 | 20706334 | C | T | 0.0212098 | -0.336807 | 0.0715567 | 2.52E-06 | 0.004709973 | 22.15448252 |
| rs144536316 | NPDR | 6 | 27466315 | A | G | 0.0289402 | 0.487884 | 0.0618647 | 3.11E-15 | 0.013378599 | 62.19383385 |
| rs170316 | NPDR | 5 | 124258600 | T | C | 0.224399 | 0.133624 | 0.0286547 | 3.11E-06 | 0.006215244 | 21.74589087 |
| rs402072 | NPDR | 19 | 46715865 | C | T | 0.139431 | -0.171971 | 0.0365208 | 2.49E-06 | 0.007097174 | 22.1732763 |
| rs74203920 | NPDR | 21 | 44294411 | T | C | 0.0368152 | 0.302109 | 0.059645 | 4.08E-07 | 0.006472829 | 25.65542662 |
| rs77324455 | NPDR | 6 | 24330507 | T | C | 0.0275188 | 0.350635 | 0.0662059 | 1.18E-07 | 0.006580384 | 28.04898447 |
| rs77681307 | NPDR | 20 | 16221961 | T | C | 0.0529317 | 0.232346 | 0.0505969 | 4.39E-06 | 0.005412495 | 21.08737796 |
| rs11047391 | PDR | 12 | 24357612 | A | G | 0.459031 | 0.072284 | 0.0157756 | 4.61E-06 | 0.002594949 | 20.99484064 |
| rs115380430 | PDR | 6 | 29881480 | C | A | 0.0239713 | 0.250778 | 0.0476112 | 1.39E-07 | 0.002942816 | 27.74345942 |
| rs12476469 | PDR | 2 | 866307 | C | T | 0.290414 | 0.0809985 | 0.017093 | 2.15E-06 | 0.002703999 | 22.45522274 |
| rs140965240 | PDR | 6 | 126597078 | T | C | 0.100941 | -0.13019 | 0.0269661 | 1.38E-06 | 0.003076388 | 23.3087494 |
| rs2069206 | PDR | 1 | 245810438 | G | A | 0.828374 | 0.097964 | 0.0214035 | 4.72E-06 | 0.002728805 | 20.9490062 |
| rs2983434 | PDR | 20 | 64138760 | T | C | 0.31329 | 0.0849318 | 0.0169076 | 5.08E-07 | 0.003103777 | 25.23345549 |
| rs3956828 | PDR | 12 | 119898249 | T | C | 0.199144 | -0.0950763 | 0.0201554 | 2.39E-06 | 0.002883342 | 22.25162343 |
| rs5769966 | PDR | 22 | 49156793 | A | G | 0.749338 | 0.0955204 | 0.0185917 | 2.78E-07 | 0.003427587 | 26.3969688 |
| rs6902545 | PDR | 6 | 33933369 | A | G | 0.356084 | 0.0870208 | 0.0163224 | 9.75E-08 | 0.003472624 | 28.42353633 |
| rs76518593 | PDR | 9 | 129587761 | G | A | 0.17751 | 0.0967031 | 0.0202837 | 1.87E-06 | 0.002730639 | 22.72931961 |
| DR and its subtypes database from IEU OpenGWAS | | | | | | | | | | | |
| rs11083103 | DR | 18 | 22541686 | G | T | 0.3608 | 0.0628 | 0.0137 | 4.59E-06 | 0.001819083 | 21.01252065 |
| rs11243147 | DR | 6 | 7268031 | T | C | 0.5601 | 0.0731 | 0.0133 | 4.30E-08 | 0.002633203 | 30.20866075 |
| rs12901288 | DR | 15 | 78267854 | C | T | 0.07467 | 0.118 | 0.0253 | 3.11E-06 | 0.001924141 | 21.75319096 |
| rs12907757 | DR | 15 | 38702126 | T | G | 0.02953 | 0.1958 | 0.0391 | 5.44E-07 | 0.002197359 | 25.07678521 |
| rs146266330 | DR | 9 | 34834134 | G | A | 0.03666 | 0.1687 | 0.0355 | 1.98E-06 | 0.002010167 | 22.58257489 |
| rs2676275 | DR | 17 | 3868171 | C | T | 0.1447 | 0.0914 | 0.019 | 1.44E-06 | 0.002067804 | 23.14116343 |
| rs4546147 | DR | 3 | 167081928 | T | G | 0.3671 | -0.0689 | 0.0137 | 5.04E-07 | 0.002205911 | 25.29282327 |
| rs4858066 | DR | 3 | 23294188 | G | A | 0.353 | 0.072 | 0.0137 | 1.54E-07 | 0.002367958 | 27.62001172 |
| rs6925683 | DR | 6 | 33894292 | G | T | 0.274 | 0.0717 | 0.0148 | 1.20E-06 | 0.002045293 | 23.47009679 |
| rs7260507 | DR | 19 | 41947625 | C | A | 0.3786 | -0.0774 | 0.0137 | 1.49E-08 | 0.002818797 | 31.91837605 |
| rs882668 | DR | 21 | 47295021 | G | A | 0.5923 | -0.0652 | 0.0134 | 1.15E-06 | 0.002053088 | 23.67476053 |
| rs115305913 | NPDR | 5 | 113528825 | T | G | 0.03407 | 0.4516 | 0.0931 | 1.23E-06 | 0.013423187 | 23.52927278 |
| rs11243147 | PDR | 6 | 7268031 | T | C | 0.5602 | 0.0921 | 0.0166 | 3.02E-08 | 0.004179724 | 30.78244303 |
| rs145526532 | PDR | 3 | 189397942 | G | A | 0.03318 | 0.2199 | 0.0465 | 2.27E-06 | 0.003102433 | 22.3637461 |
| rs4502225 | PDR | 16 | 78424831 | C | T | 0.8738 | -0.1201 | 0.0247 | 1.19E-06 | 0.003181174 | 23.64242981 |
| rs7260507 | PDR | 19 | 41947625 | C | A | 0.3789 | -0.0785 | 0.017 | 3.88E-06 | 0.002900384 | 21.32266436 |

SNP, single nucleotide polymorphism; Chr, chromosome; Pos, position; EAF, Effect allele frequency; SE, standard error; DR, diabetic retinopathy; NPDR, background diabetic retinopathy; PDR, proliferative diabetic retinopathy.

**Table S2. Characteristics of selected SNPs for AD**

| **SNP** | **Trait** | **Chr** | **Pos.** | **Effect allele** | **Other allele** | **EAF** | **Beta** | **SE** | **P value** | **R^2^** | **F statistic** |
| --- | --- | --- | --- | --- | --- | --- | --- | --- | --- | --- | --- |
| rs10091076 | AD | 8 | 102571882 | C | T | 0.30962 | 0.011760749 | 0.002336996 | 4.84E-07 | 5.91E-05 | 25.32526652 |
| rs11100203 | AD | 4 | 158936387 | G | A | 0.36392 | -0.01036925 | 0.002251937 | 4.13E-06 | 4.98E-05 | 21.20226301 |
| rs11218343 | AD | 11 | 121564878 | C | T | 0.044072 | -0.035926385 | 0.005255086 | 8.12E-12 | 1.09E-04 | 46.73770509 |
| rs11257238 | AD | 10 | 11675398 | C | T | 0.36096 | 0.012943374 | 0.002261352 | 1.04E-08 | 7.73E-05 | 32.76111405 |
| rs117264457 | AD | 19 | 44901175 | A | G | 0.014922 | -0.043406507 | 0.009447807 | 4.34E-06 | 5.54E-05 | 21.10802752 |
| rs117285649 | AD | 10 | 42722997 | C | T | 0.035679 | 0.027869782 | 0.005813546 | 1.64E-06 | 5.34E-05 | 22.98184636 |
| rs12593078 | AD | 15 | 63225148 | A | G | 0.38240 | -0.011578284 | 0.002224094 | 1.93E-07 | 6.33E-05 | 27.10080049 |
| rs143332484 | AD | 6 | 41161469 | T | C | 0.0074684 | 0.073707877 | 0.013303944 | 3.02E-08 | 8.05E-05 | 30.69495412 |
| rs143465942 | AD | 16 | 85700458 | A | G | 0.0095011 | 0.057510674 | 0.011826655 | 1.16E-06 | 6.23E-05 | 23.64683705 |
| rs17646025 | AD | 15 | 50727615 | T | C | 0.18195 | -0.013594433 | 0.002795737 | 1.16E-06 | 5.50E-05 | 23.64446921 |
| rs1859788 | AD | 7 | 100374211 | A | G | 0.3243 | -0.018395737 | 0.002312745 | 1.80E-15 | 1.48E-04 | 63.26723904 |
| rs204473 | AD | 19 | 44984262 | A | G | 0.02438 | -0.041669554 | 0.006996017 | 2.58E-09 | 8.26E-05 | 35.47610993 |
| rs2081545 | AD | 11 | 60190907 | A | C | 0.38212 | -0.017870026 | 0.002229747 | 1.11E-15 | 1.51E-04 | 64.23018786 |
| rs2544702 | AD | 5 | 87152123 | A | C | 0.24952 | -0.012000649 | 0.002497332 | 1.54E-06 | 5.39E-05 | 23.09175297 |
| rs28636611 | AD | 4 | 112061367 | T | G | 0.29335 | 0.012013557 | 0.002385209 | 4.74E-07 | 5.98E-05 | 25.36824059 |
| rs35283920 | AD | 16 | 70653292 | T | C | 0.31300 | 0.012430612 | 0.002336435 | 1.04E-07 | 6.65E-05 | 28.30594217 |
| rs3752229 | AD | 19 | 1041353 | G | A | 0.050924 | 0.024779708 | 0.004905081 | 4.38E-07 | 5.94E-05 | 25.521136 |
| rs3865444 | AD | 19 | 51224706 | A | C | 0.29928 | -0.013757048 | 0.002354744 | 5.15E-09 | 7.94E-05 | 34.13210769 |
| rs4311 | AD | 17 | 63483402 | T | C | 0.47880 | -0.011344778 | 0.002173127 | 1.78E-07 | 6.42E-05 | 27.25346889 |
| rs442495 | AD | 15 | 58730416 | C | T | 0.35434 | -0.013721107 | 0.002257715 | 1.22E-09 | 8.61E-05 | 36.93516686 |
| rs6014724 | AD | 20 | 56423488 | G | A | 0.095042 | -0.022894258 | 0.00368812 | 5.38E-10 | 9.02E-05 | 38.53390824 |
| rs6125601 | AD | 20 | 37611619 | A | G | 0.22596 | 0.0119702 | 0.002605802 | 4.36E-06 | 5.01E-05 | 21.10182355 |
| rs6448453 | AD | 4 | 11024404 | A | G | 0.26247 | 0.014704637 | 0.002450874 | 1.98E-09 | 8.37E-05 | 35.99702806 |
| rs6499775 | AD | 16 | 55725955 | G | A | 0.13605 | 0.014651 | 0.003149776 | 3.30E-06 | 5.05E-05 | 21.63591157 |
| rs6890748 | AD | 5 | 154308540 | A | G | 0.4162 | 0.011090077 | 0.002195832 | 4.41E-07 | 5.98E-05 | 25.50767668 |
| rs7149638 | AD | 14 | 52880213 | T | C | 0.10437 | 0.016752748 | 0.003533267 | 2.12E-06 | 5.25E-05 | 22.48118488 |
| rs76953845 | AD | 11 | 5335981 | G | T | 0.042917 | 0.027747243 | 0.005667806 | 9.80E-07 | 6.32E-05 | 23.9667818 |
| rs8003201 | AD | 14 | 23008384 | A | G | 0.12375 | 0.015307262 | 0.003286574 | 3.20E-06 | 5.08E-05 | 21.69242981 |
| rs846881 | AD | 19 | 44575305 | C | A | 0.20705 | -0.01736942 | 0.002685173 | 9.89E-11 | 9.91E-05 | 41.84331562 |
| rs867611 | AD | 11 | 86065502 | G | A | 0.31742 | -0.020428916 | 0.002323835 | 1.48E-18 | 1.81E-04 | 77.2823053 |
| rs9381563 | AD | 6 | 47464901 | C | T | 0.35574 | 0.0144515 | 0.002271607 | 1.99E-10 | 9.57E-05 | 40.47244652 |
| rs12590654 | AD | 14 | 92472511 | A | G | 0.3365 | -0.014826611 | 0.002307804 | 1.32E-10 | 9.82E-05 | 41.27489176 |
| rs111278892 | AD | 19 | 1039323 | G | C | 0.14714 | 0.019911604 | 0.003050251 | 6.67E-11 | 9.95E-05 | 42.61292411 |
| rs1171812 | AD | 10 | 61655297 | C | T | 0.47519 | 0.010222663 | 0.002175097 | 2.60E-06 | 5.21E-05 | 22.08873421 |
| rs4817090 | AD | 21 | 27534261 | C | T | 0.36143 | 0.011508897 | 0.002273862 | 4.16E-07 | 6.11E-05 | 25.61764475 |
| rs6910948 | AD | 6 | 22306698 | C | A | 0.25722 | 0.013054194 | 0.002478636 | 1.39E-07 | 6.51E-05 | 27.73796646 |
| rs9401134 | AD | 6 | 98199541 | C | G | 0.088605 | 0.017853041 | 0.003817652 | 2.92E-06 | 5.15E-05 | 21.86914125 |
| rs10933431 | AD | 2 | 233981912 | G | C | 0.24449 | -0.015436971 | 0.00250901 | 7.62E-10 | 8.80E-05 | 37.85466368 |
| rs3859570 | AD | 19 | 18510925 | C | T | 0.41618 | 0.026392498 | 0.005358658 | 8.43E-07 | 3.38E-04 | 24.25766051 |
| rs679515 | AD | 1 | 207750568 | T | C | 0.17148 | 0.025417699 | 0.00286314 | 6.83E-19 | 1.84E-04 | 78.8110831 |

SNP, single nucleotide polymorphism; Chr, chromosome; Pos, position; EAF, Effect allele frequency; SE, standard error, AD, alzheimer's disease.

**Table S3 Sensitivity analysis of the associations between AD and DR**

| **Exposure** | **Outcome** | **IVW**  **OR (95% CI)**  **P value** | **MR-Egger**  **OR (95% CI)**  **P value** | **Weighted median**  **OR (95% CI)**  **P value** | **Simple mode**  **OR (95% CI)**  **P value** | **Weighted mode**  **OR (95% CI)**  **P value** |
| --- | --- | --- | --- | --- | --- | --- |
| DR and its subtypes database from FinnGen consortium | | | | | | |
| AD | DR | 2.5090 (1.2102, 5.2018)  0.0134 | 2.3060 (0.3346, 5.8903)  0.4068 | 1.7463 (0.6576, 4.6371)  0.2631 | 1.1744 (0.1967, 7.0111)  0.8618 | 1.2652 (0.2176, 7.3563)  0.7961 |
|  | NPDR | 2.7455 (1.3178, 5.7197)  0.0069 | 1.2911 (0.1415, 4.7780)  0.8224 | 2.3501 (0.8403, 6.5728)  0.1034 | 1.2462 (0.1691, 9.1827)  0.8305 | 1.1435 (0.1994, 6.5568)  0.8814 |
|  | PDR | 2.3098 (1.2411, 4.2986)  0.0082 | 1.0993 (0.1038, 6.6464)  0.9383 | 2.1420 (0.9378, 4.8921)  0.0707 | 1.4348 (0.4125, 4.9906)  0.5773 | 2.5464 (0.6940, 9.3430)  0.1758 |
| DR and its subtypes database from IEU OpenGWAS | | | | | | |
| AD | DR | 1.9263 (1.2418, 2.9882)  0.0034 | 1.1705 (0.3234, 4.2364)  0.8123 | 1.1994 (0.6622, 2.1725)  0.5486 | 1.0019 (0.3335, 3.0096)  0.9974 | 1.0538 (0.3743, 2.9668)  0.9217 |
|  | NPDR | 2.8233 (1.1916, 6.6892)  0.0184 | 1.1331 (0.0921, 3.9414)  0.9228 | 1.5778 (0.4428, 5.6214)  0.4818 | 1.2922 (0.1292, 9.6961)  0.8286 | 1.3811 (0.2766, 6.8969)  0.6964 |
|  | PDR | 1.9535 (1.1622, 3.2834)  0.0115 | 1.0120 (0.2258, 4.5359)  0.9877 | 1.5540 (0.7506, 3.2173)  0.2350 | 1.3229 (0.3039, 5.7581)  0.7118 | 1.3520 (0.3920, 4.6622)  0.6365 |
| DR and its subtypes database from FinnGen consortium | | | | | | |
| DR | AD | 1.0144 (1.0028, 1.0261)  0.0150 | 1.0310 (1.0050, 1.0578)  0.0299 | 1.0083 (0.9935, 1.0231)  0.2698 | 1.0070 (0.9822, 1.0324)  0.5870 | 1.0061 (0.9841, 1.0283)  0.5927 |
| NPDR |  | 1.0247 (1.0083, 1.0415)  0.0031 | 1.0180 (0.9819, 1.0555)  0.3869 | 1.0262 (1.0044, 1.0484)  0.0179 | 1.0234 (0.9932, 1.0544)  0.1904 | 1.0268 (0.9989, 1.0554)  0.1182 |
| PDR |  | 1.0413 (1.0150, 1.0684)  0.0020 | 1.0181 (0.8736, 1.1864)  0.8243 | 1.0280 (0.9929, 1.0642)  0.1189 | 1.0274 (0.9791, 1.0780)  0.2990 | 1.0266 (0.9847, 1.0703)  0.2476 |
| DR and its subtypes database from IEU OpenGWAS | | | | | | |
| DR | AD | 1.0240 (1.0041, 1.0443)  0.0176 | 1.0002 (0.9304, 1.0752)  0.9955 | 1.0202 (0.9947, 1.0462)  0.1207 | 1.0104 (0.9732, 1.0498)  0.5992 | 1.0132 (0.9763, 1.0516)  0.5038 |
| NPDR |  | 1.0347 (1.0000, 1.0706)  0.0497 | - | - | - | - |
| PDR |  | 1.0568 (1.0230, 1.0918)  0.0008 | 1.0115 (0.8544, 1.1975)  0.9062 | 1.0442 (0.9999, 1.0904)  0.0502 | 1.0264 (0.9699, 1.0861)  0.4335 | 1.0400 (0.9892, 1.0933)  0.2218 |

IVW, inverse-variance weighted; DR, diabetic retinopathy; NPDR, background diabetic retinopathy; PDR, proliferative diabetic retinopathy; AD, alzheimer's disease.

**Table S4. Power calculation for two-sample MR analysis**

| **Exposure** | **Outcome** | **Sample size** | **Proportion of cases** | **OR** | **R^2^** | **Power** |
| --- | --- | --- | --- | --- | --- | --- |
| DR and its subtypes database from FinnGen consortium | | | | | | |
| AD | DR | 320,030 | 0.01871075 | 2.5090 | 0.001467354 | 99% |
|  | NPDR | 317,488 | 0.01085395 | 2.7455 | 0.0023926 | 100% |
|  | PDR | 338,139 | 0.02479158 | 2.3098 | 0.00141202 | 99% |
| DR and its subtypes database from IEU OpenGWAS | | | | | | |
| AD | DR | 190,594 | 0.076518673 | 1.9263 | 0.001925062 | 99% |
|  | NPDR | 206,234 | 0.009823792 | 2.8233 | 0.00300864 | 99% |
|  | PDR | 212,889 | 0.040777119 | 1.9535 | 0.002139936 | 98% |
| DR and its subtypes database from FinnGen consortium | | | | | | |
| DR | AD | 455,258 | 0.157888494 | 1.0144 | 0.101936857 | 20% |
| NPDR |  | 455,258 | 0.157888494 | 1.0247 | 0.045156726 | 25% |
| PDR |  | 455,258 | 0.157888494 | 1.0413 | 0.029664926 | 41% |
| DR and its subtypes database from IEU OpenGWAS | | | | | | |
| DR | AD | 455,258 | 0.157888494 | 1.0240 | 0.024142803 | 15% |
| NPDR |  | 455,258 | 0.157888494 | 1.0347 | 0.013423187 | 17% |
| PDR |  | 455,258 | 0.157888494 | 1.0568 | 0.013363715 | 36% |

MR, Mendelian randomization; OR, odds ratio; Type-I error rate (α) was set as 0.05; DR, diabetic retinopathy; PDR, proliferative diabetic retinopathy; NPDR, background diabetic retinopathy; AD, alzheimer's disease; nSNPs, number of single-nucleotide polymorphisms;





**Fig. S1** The leave-one-out sensitivity analysis. The leave-one-out analysis for AD to DR (FinnGen) (A), NPDR (FinnGen) (B), PDR (FinnGen) (C), DR (IEU) (D), NPDR (IEU) (E), PDR (IEU) (F). The leave-one-out sensitivity analysis for DR (FinnGen) (G), NPDR (FinnGen) (H), PDR (FinnGen) (I), DR (IEU) (J), PDR (IEU) (K) to AD.


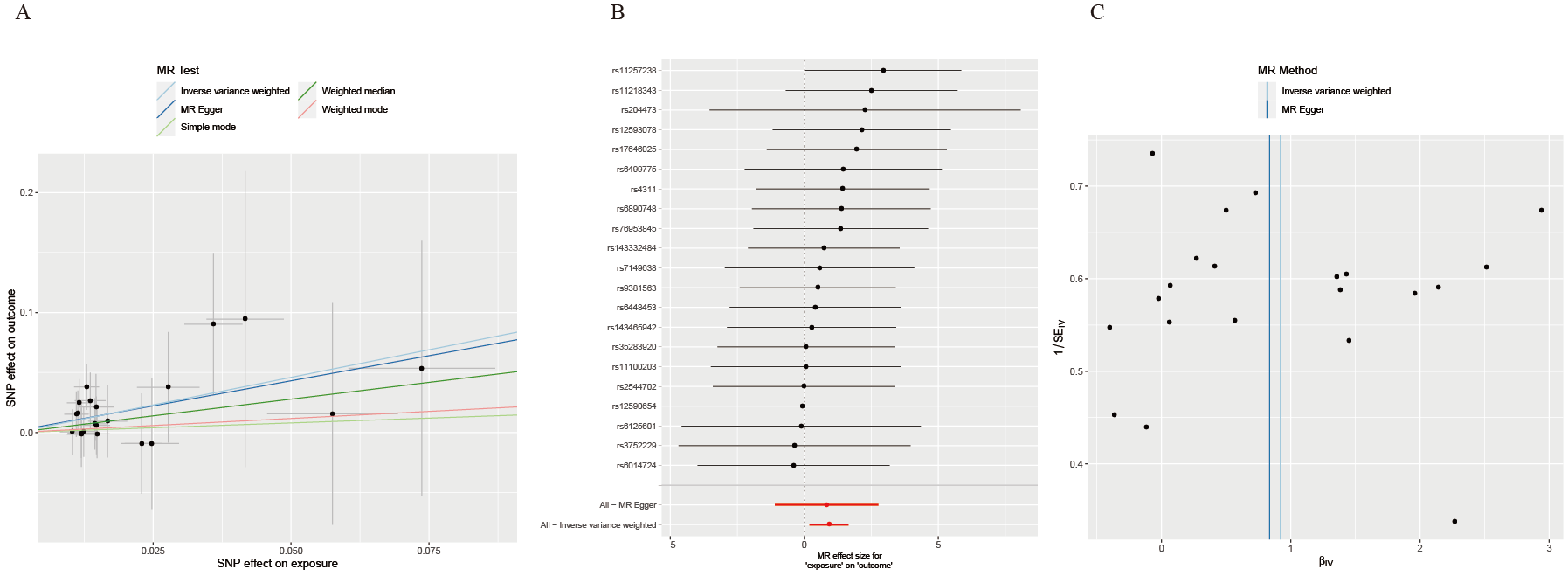


**Fig. S2** The causal effect of AD on DR (FinnGen). (A) Scatter plot, (B) Forest plot, (C) Funnel plot.


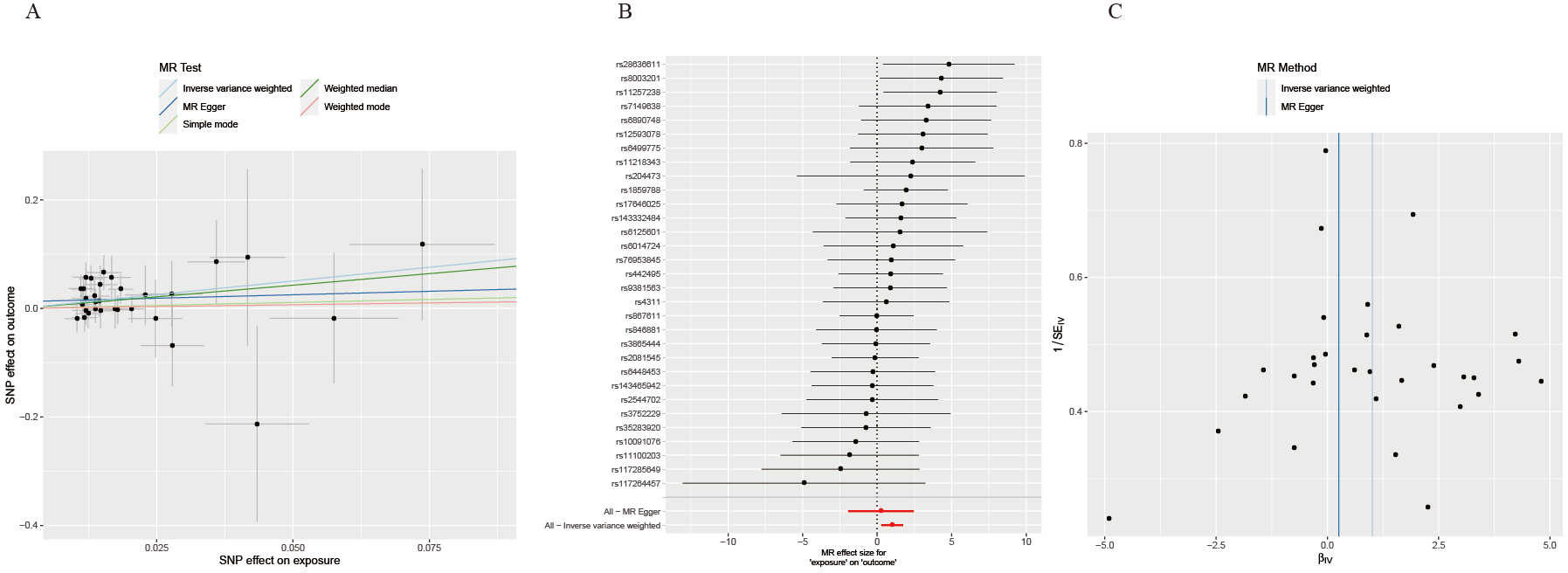


**Fig. S3** The causal effect of AD on NPDR (FinnGen). (A) Scatter plot, (B) Forest plot, (C) Funnel plot.


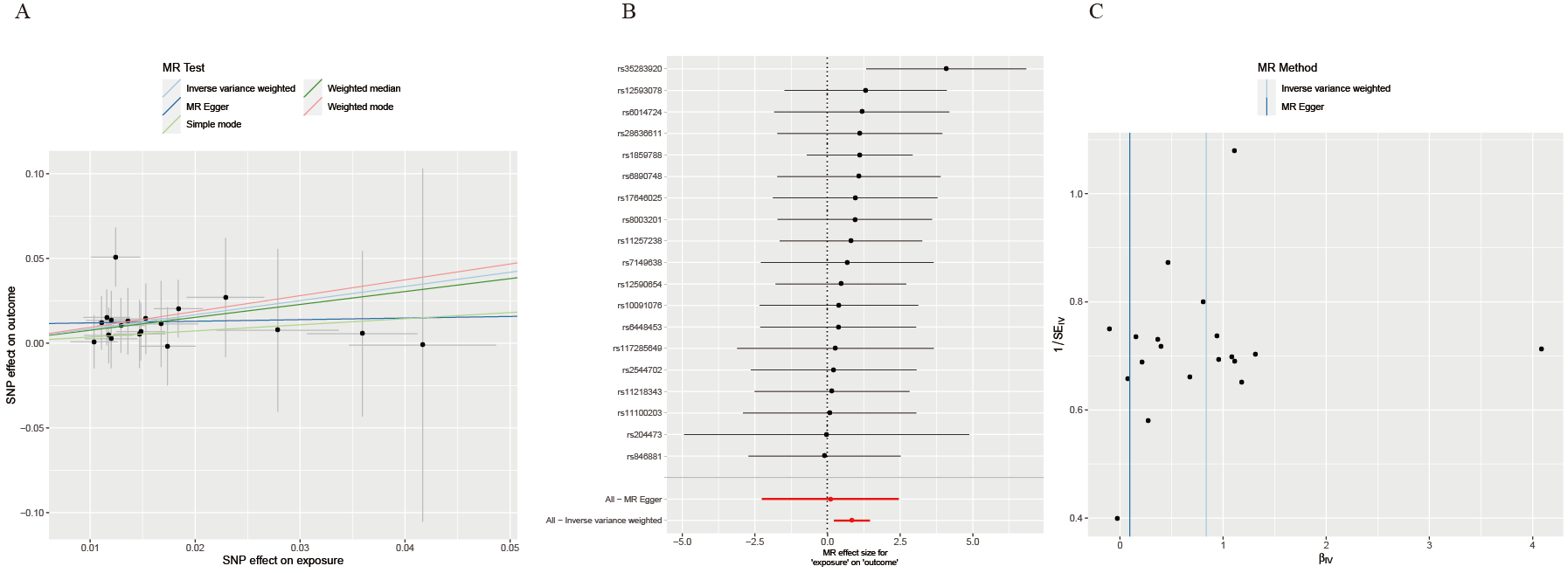


**Fig. S4** The causal effect of AD on PDR (FinnGen). (A) Scatter plot, (B) Forest plot, (C) Funnel plot.


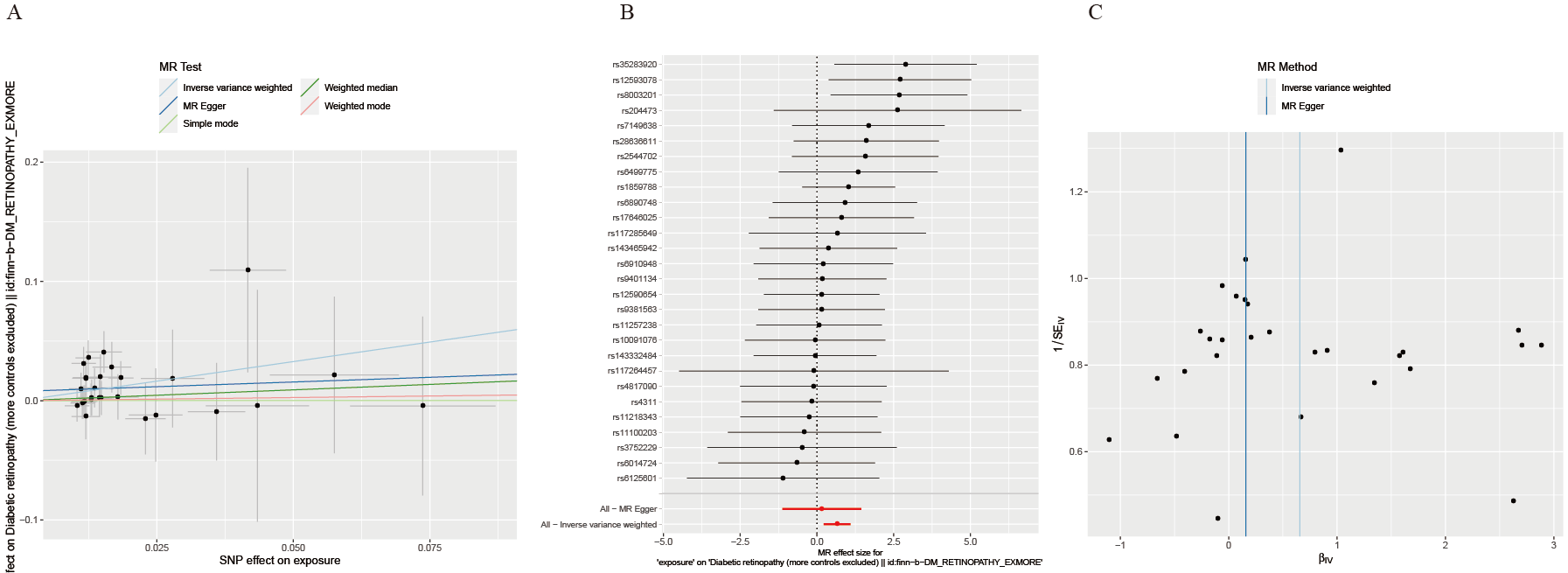


**Fig. S5** The causal effect of AD on DR (IEU). (A) Scatter plot, (B) Forest plot, (C) Funnel plot.


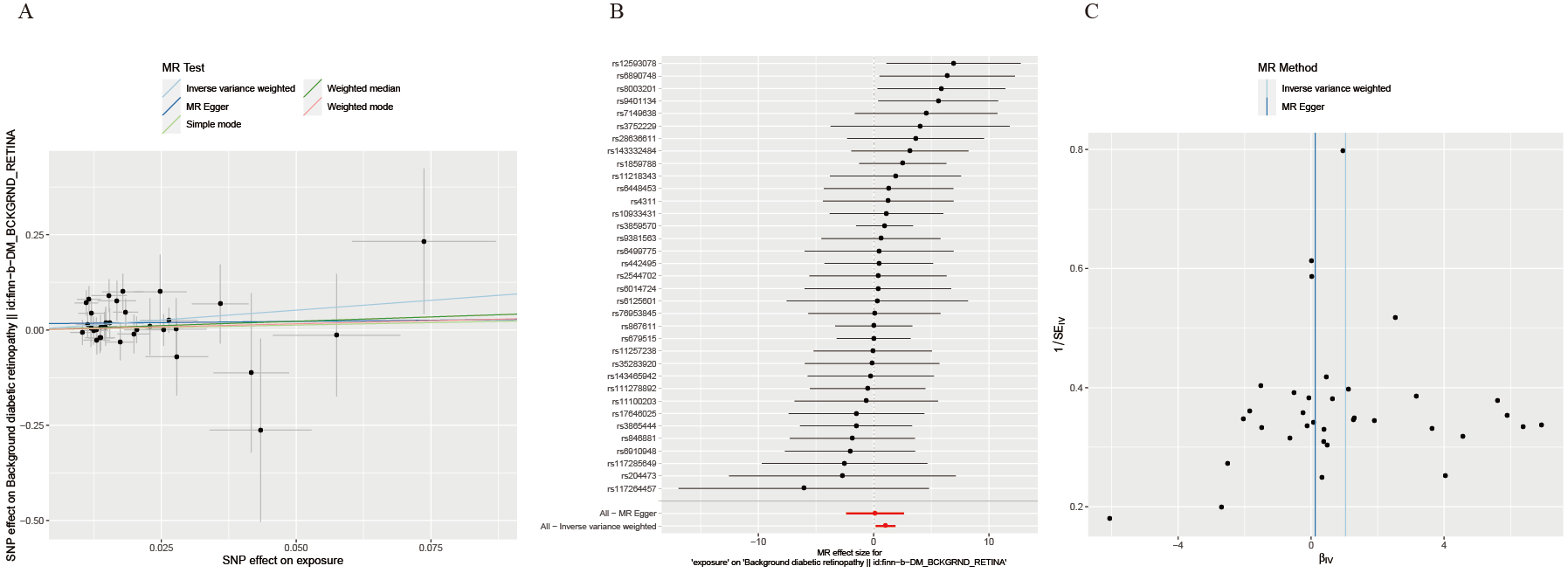


**Fig. S6** The causal effect of AD on NPDR (IEU). (A) Scatter plot, (B) Forest plot, (C) Funnel plot.


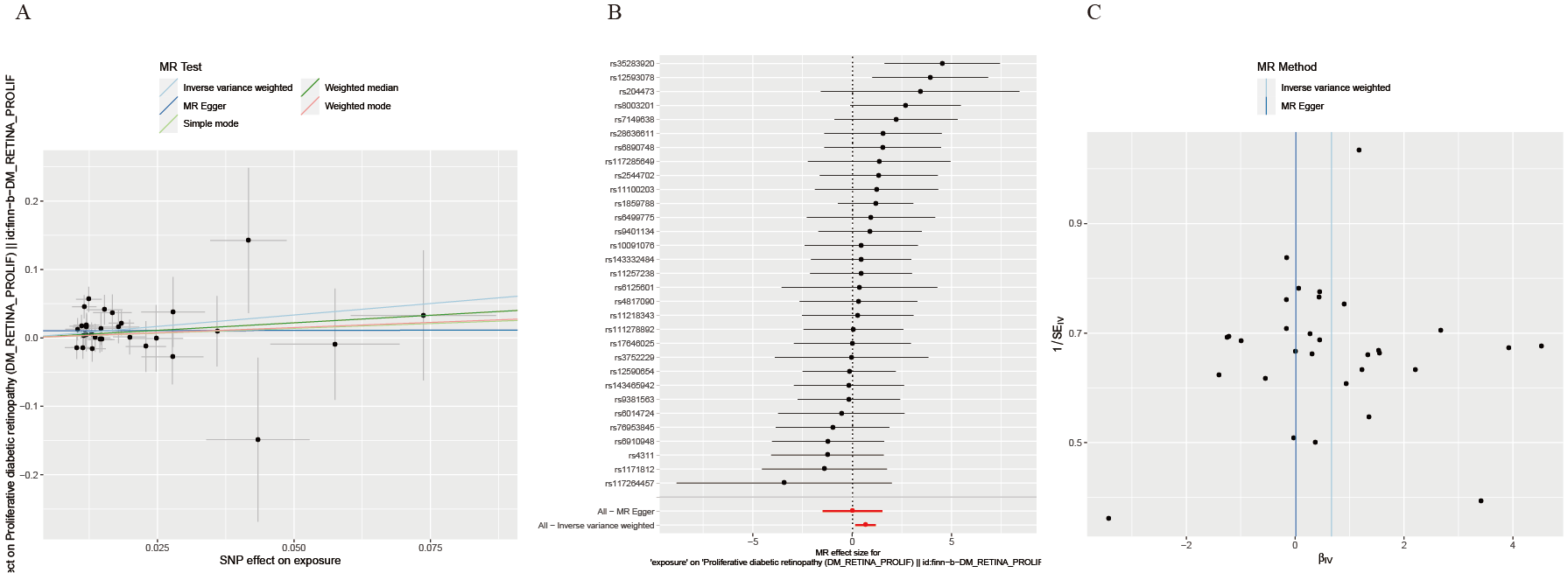


**Fig. S7** The causal effect of AD on PDR (IEU). (A) Scatter plot, (B) Forest plot, (C) Funnel plot.


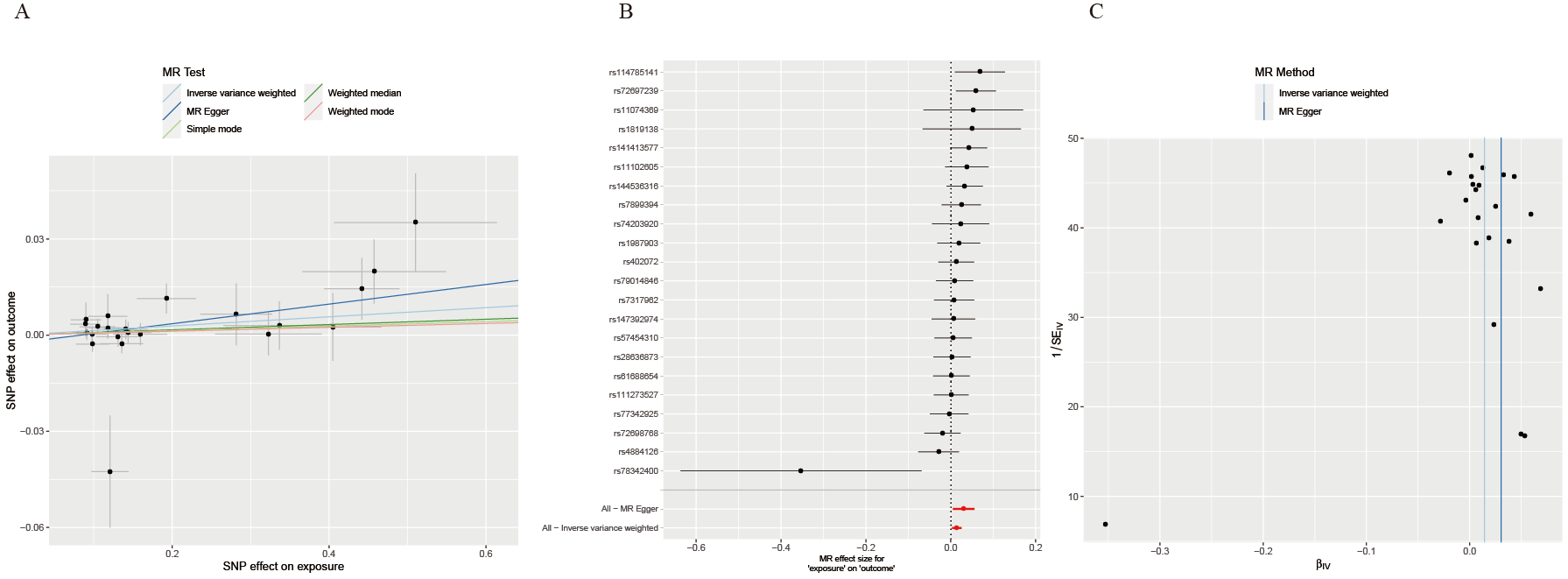


**Fig. S8** The causal effect of DR (FinnGen) on AD. (A) Scatter plot, (B) Forest plot, (C) Funnel plot.


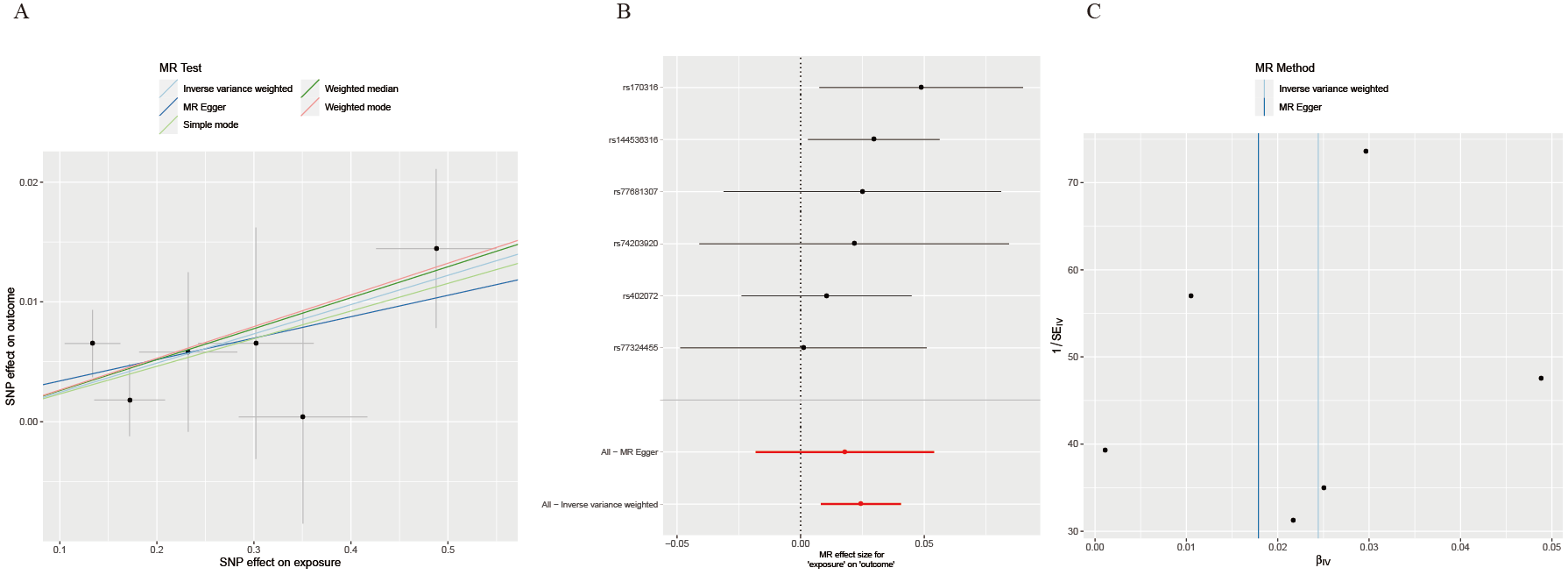


**Fig. S9** The causal effect of NPDR (FinnGen) on AD. (A) Scatter plot, (B) Forest plot, (C) Funnel plot.


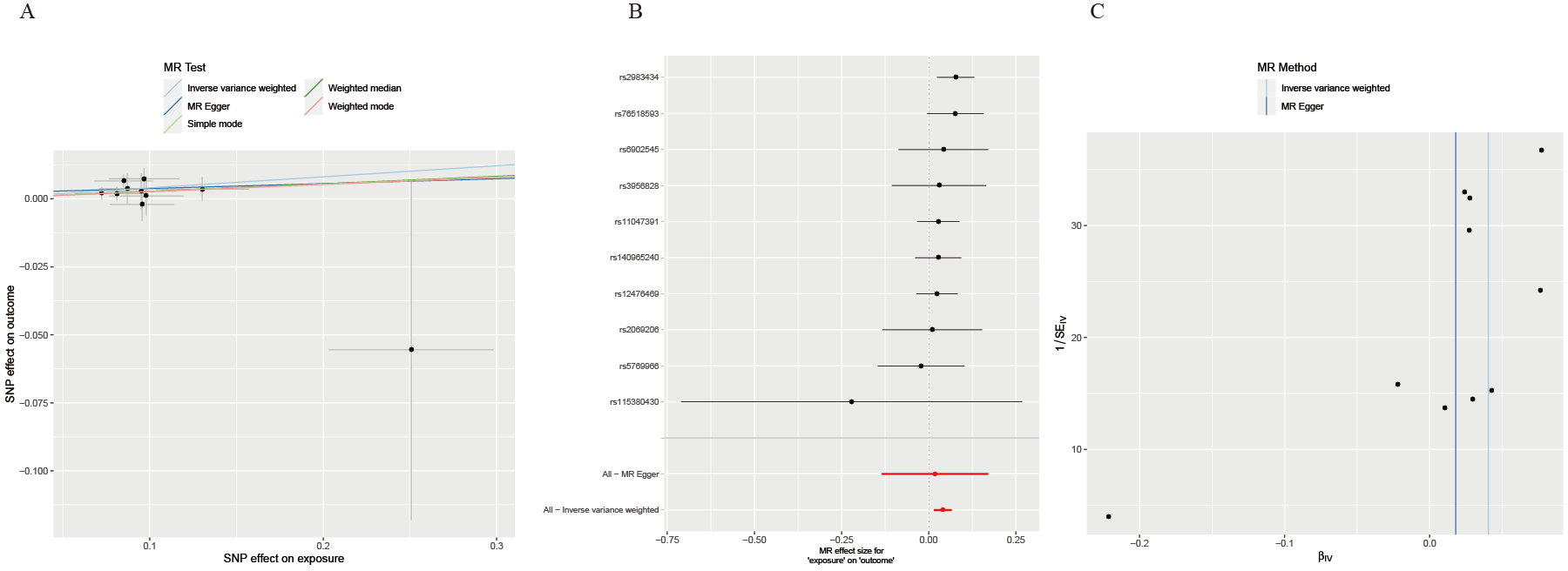


**Fig. S10** The causal effect of PDR (FinnGen) on AD. (A) Scatter plot, (B) Forest plot, (C) Funnel plot.


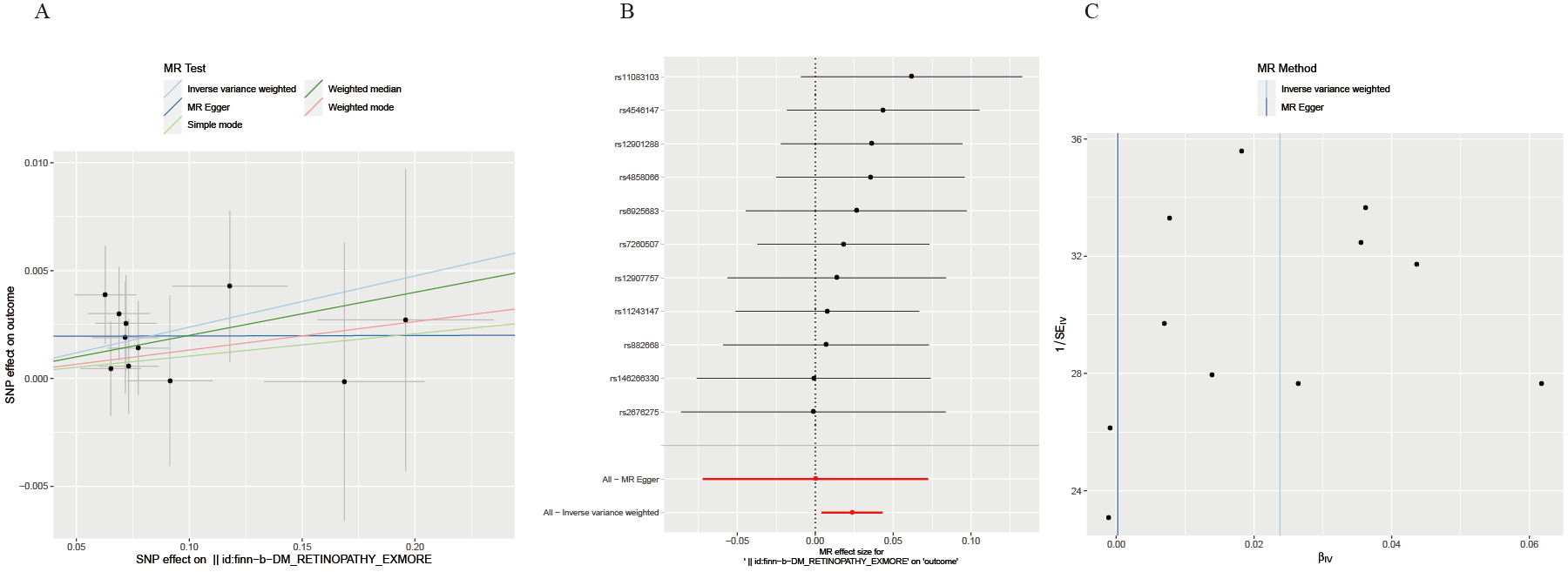


**Fig. S11** The causal effect of DR (IEU) on AD. (A) Scatter plot, (B) Forest plot, (C) Funnel plot.


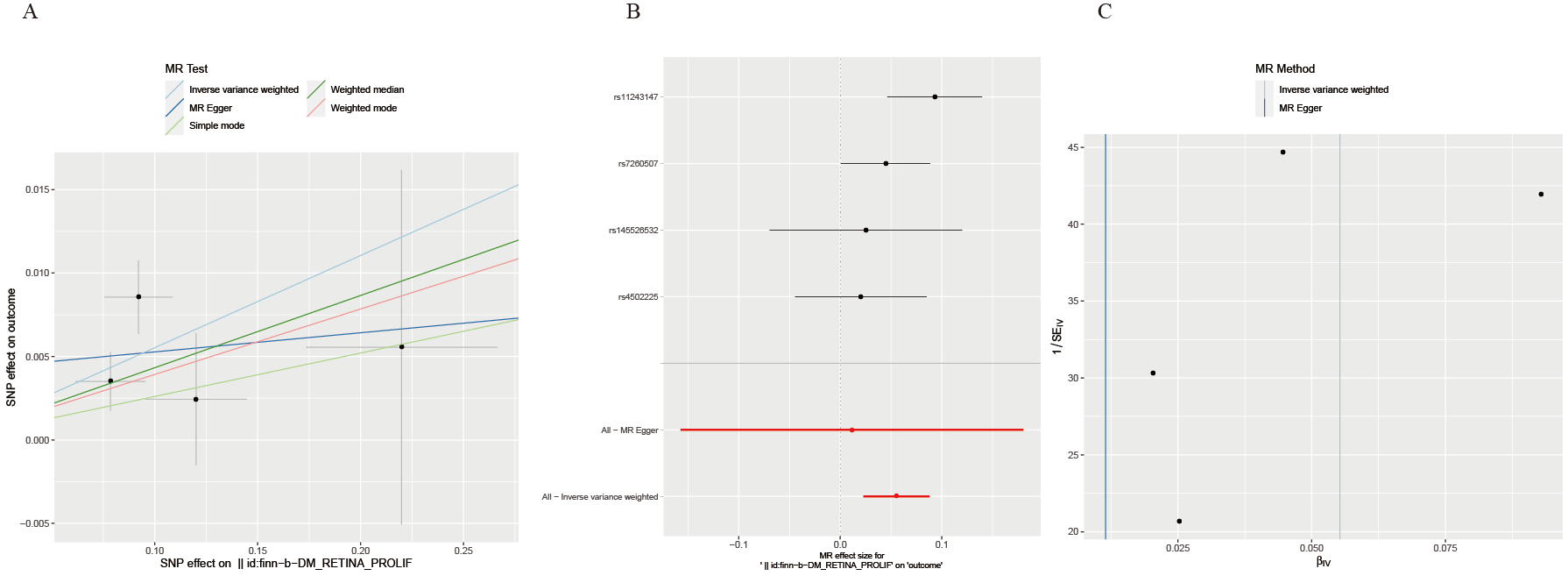


**Fig. S12** The causal effect of PDR (IEU) on AD. (A) Scatter plot, (B) Forest plot, (C) Funnel plot.


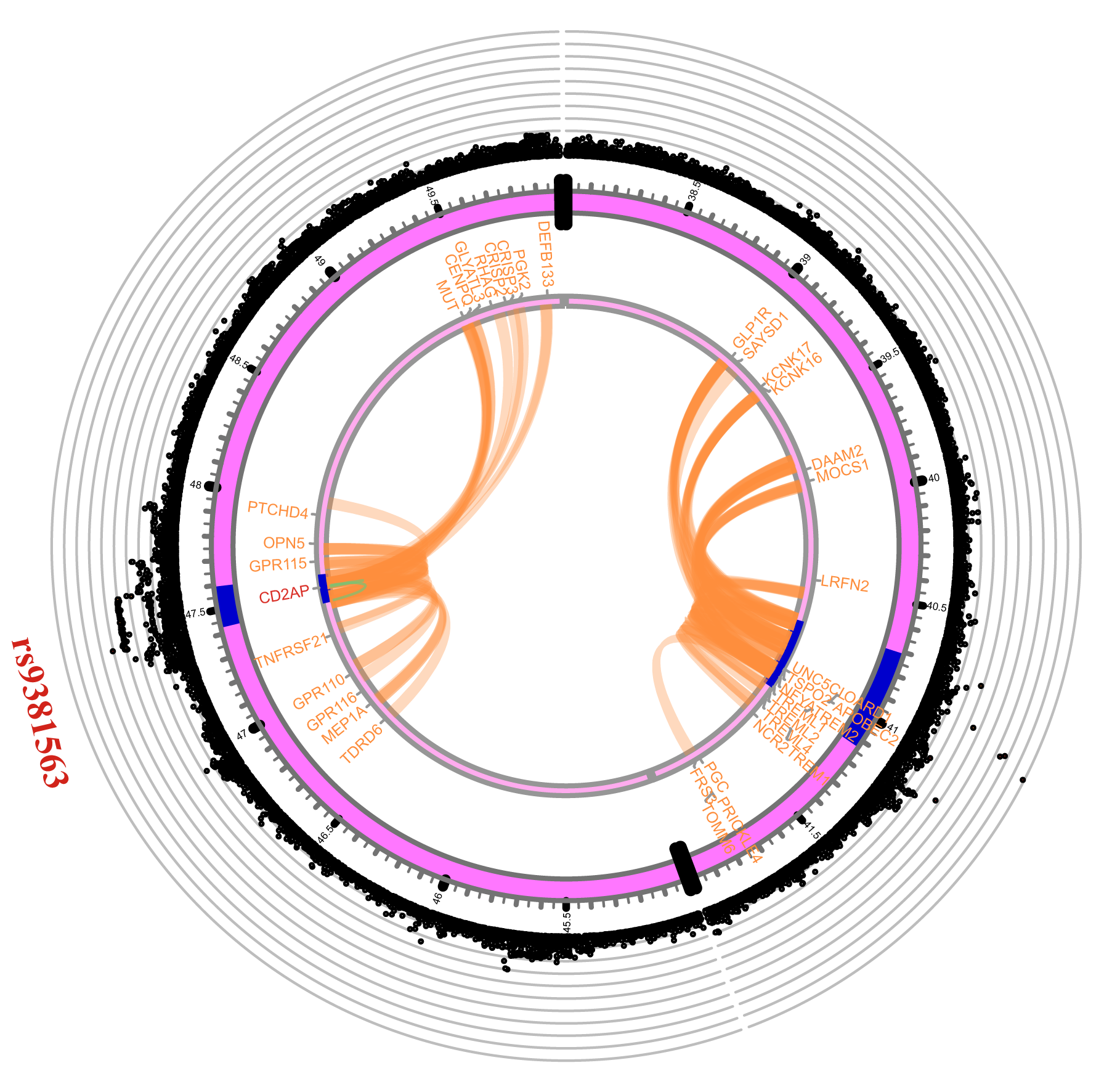


#### **Fig. S13** Circos plots of chromatin interactions and eQTLs of Chromosome 6. The outer circle of the GWAS plot displays SNP associations (grey circles) with -log10 (p-value). In the Manhattan plot, only SNPs with P<0.05 are displayed. SNPs in genomic risk loci are color-coded based on their maximum r^2^ to the independent significant SNPs in the locus, with red (r^2^>0.8), orange (r^2^>0.6), green (r^2^>0.4), and blue (r^2^>0.2). SNPs that are not in linkage disequilibrium with any of the independent significant SNPs (with r^2^≤0.2) are gray. The chromosome ring, the second layer of the Circos plot, highlights genomic risk loci in blue. The third layer displays mapped genes by chromatin interactions or eQTLs, colored orange or green, respectively. The same is true of the second layer, but without coordinates to align the position of genes with genomic coordinate. Links colored orange represent chromatin interactions, green are eQTLs, and blue if both a chromatin interaction and an eQTL.
