# Supplementary material for: Alzheimer’s disease as a causal risk factor for diabetic retinopathy: a Mendelian randomization study
Source: Front Endocrinol (Lausanne). 2024 Apr 18;15:1340608. doi: 10.3389/fendo.2024.1340608 (PMC11064697; doi:10.3389/fendo.2024.1340608)
Supplement: Supplementary file 1 [file DataSheet_1.zip › MR Code.doc]

1. **two-sample MR**

library(TwoSampleMR)

library(data.table)

library(MRPRESSO)

setwd("C:/Users")

Exposure<-read.delim("Exposure.txt", sep = "\t", header = T)

Outcome<-fread("Outcome.txt")

Exposure_p<-subset(Exposure, p<5e-08)

write.csv(Exposure_p, file="Exposure_p.csv")

Exposure_exp<-read.csv("Exposure_p.csv",header = T)

Exposure_exp<-format_data(Exposure_exp,type='exposure',snp_col = "SNP",beta_col = "beta",se_col = "se",effect_allele_col = "A1",other_allele_col = "A2",eaf_col="eaf",pval_col = "p")

Exposure_exp_clumped<-clump_data(Exposure_exp,clump_kb=5000,clump_r2=0.001,clump_p1=1,clump_p2=1,pop="EUR")

files <- list.files(pattern = "\\.txt$")

for(file in files) {

data_name <- sub("\\.txt$", "", file)

data <- fread(file)

merged_data <- merge(Exposure_clumped, data, by.x = "SNP", by.y = "MarkerName")

merge_file_name <- paste0( data_name, "_merge.csv")

write.csv(merged_data, merge_file_name, row.names = FALSE)

outcome_data <- read_outcome_data(

snps = Exposure_clumped$SNP,

filename = merge_file_name,

sep = ",",

snp_col = "SNP",

beta_col = "Effect",

se_col = "StdErr",

effect_allele_col = "Allele1",

other_allele_col = "Allele2",

pval_col = "P-vallue",

eaf_col = "Freq1"

)

out_file_name <- paste0(data_name, "_out.csv")

write.csv(outcome_data, out_file_name, row.names = FALSE)

harmonised_data <- harmonise_data(

exposure_dat = Exposure_clumped,

outcome_dat = outcome_data,

action = 3

)

harmonised_data <- harmonised_data[harmonised_data$palindromic != TRUE, ]

har_file_name <- paste0("har_", data_name, ".csv")

write.csv(harmonised_data, har_file_name, row.names = FALSE)

}

#MR-PRESSO

files <- list.files(pattern = "^har_.*\\.csv$")

for(file in files) {

dat <- read.csv(file, stringsAsFactors = FALSE)

result <- mr_presso(

BetaOutcome = "beta.outcome",

BetaExposure = "beta.exposure",

SdOutcome = "se.outcome",

SdExposure = "se.exposure",

OUTLIERtest = TRUE,

DISTORTIONtest = TRUE,

data = dat,

NbDistribution = 1000,

SignifThreshold = 0.05

)

data_name <- sub("^har_", "", file)

data_name <- sub("\\.csv$", "", data_name)

save_name <- paste0("Presso_", data_name, ".csv")

write.csv(result, save_name, row.names = FALSE)

}

har_files <- list.files(pattern = ".csv$")

for(file in har_files) {

dat <- read.csv(file, stringsAsFactors = FALSE)

res <- mr(dat)

OR <- generate_odds_ratios(res)

data_name <- sub("\\.csv$", "", file)

write.csv(OR, paste0("OR_", data_name, ".csv"), row.names = FALSE)

het <- mr_heterogeneity(dat)

write.csv(het, paste0("het_", data_name, ".csv"), row.names = FALSE)

pleio <- mr_pleiotropy_test(dat)

write.csv(pleio, paste0("pleio_", data_name, ".csv"), row.names = FALSE)

}

\# mr_leaveoneout_plot

dat<-read.csv("har_data.csv",header = T)

single <- mr_leaveoneout(dat)

p <- mr_leaveoneout_plot(single)

#mr_scatter_plot

mr_scatter_plot(res,dat)

#mr_forest_plot

res_single <- mr_singlesnp(dat)

mr_forest_plot(res_single)

#mr_funnel_plot

mr_funnel_plot(res_single)

2. **GSMR**

install.packages("pbapply")

install.packages("data.table")

install.packages("readxl")

install.packages("survey")

install.packages("https://yanglab.westlake.edu.cn/software/gsmr/static/gsmr_1.1.1.tar.gz",repos=NULL,type="source")

library(gsmr)

library(TwoSampleMR)

library(data.table)

library(readxl)

library(pbapply)

file_path <- "C:/Users"

all_files <- list.files(file_path, full.names = TRUE, pattern = ".csv$")

results_list <- list()

all_gsmr_IS <- list()

for(file in all_files) {

mydata <- fread(file, header = TRUE)

LD_IS <- ld_matrix(mydata$SNP, with_alleles = FALSE)

gsmr_IS <- gsmr(bzx=mydata$beta.exposure, bzx_se=mydata$se.exposure, bzx_pval=mydata$pval.exposure,

bzy=mydata$beta.outcome, bzy_se=mydata$se.outcome, bzy_pval=mydata$pval.outcome,

ldrho=LD_IS, snpid=as.character(mydata$SNP), n_ref=503, gwas_thresh=1e-5, nsnps_thresh=3, heidi_outlier_flag=T)

result <- data.frame(exposure = "HI", Outcome = "adj",

pval = gsmr_IS$bxy_pval,

or = sprintf("%0.2f", exp(gsmr_IS$bxy)),

ci = paste(sprintf("%0.2f", exp(gsmr_IS$bxy - 1.96 * gsmr_IS$bxy_se)), "-",

sprintf("%0.2f", exp(gsmr_IS$bxy + 1.96 * gsmr_IS$bxy_se)), sep = ""))

results_list[[file]] <- result

all_gsmr_IS[[file]] <- gsmr_IS

}

final_results <- do.call(rbind, results_list)

final_gsmr <- do.call(rbind, all_gsmr_IS)

head(final_results)

head(final_gsmr)

write.csv(final_results, file="final_results.csv")

write.csv(final_gsmr, file="final_gsmr.csv")

3. **MR-steiger**

library(TwoSampleMR)

dat<-read.csv("data.csv")

dat$r.exposure <- get_r_from_bsen(b = dat$beta.exposure, se = dat$se.exposure, n = dat$samplesize.exposure)

dat$r.outcome <- get_r_from_bsen(b = dat$beta.outcome, se = dat$se.outcome, n = dat$samplesize.outcome)

all_results = directionality_test(dat)

head(all_results)

filtered_results = steiger_filtering(dat)

head(filtered_results)

write.csv(filtered_results,file="filtering_har_EA_AD.csv")

single_snp_result = directionality_test(dat[2,])

head(single_snp_result)
